# Polysorbates degrading enzymes in biotherapeutics – a current status and future perspectives

**DOI:** 10.3389/fbioe.2024.1490276

**Published:** 2025-01-10

**Authors:** Marius Nicolaus Felix, Thomas Waerner, Daniel Lakatos, Bernd Reisinger, Simon Fischer, Patrick Garidel

**Affiliations:** ^1^ Analytical Development Biologicals, Boehringer Ingelheim Pharma GmbH & Co., KG, Innovation Unit, Biberach an der Riss, Germany; ^2^ Bioprocess Development Biologicals, Boehringer Ingelheim Pharma GmbH & Co., KG, Innovation Unit, Biberach an der Riss, Germany; ^3^ Pharmaceutical Development Biologicals, TIP, Boehringer Ingelheim Pharma GmbH & Co., KG, Innovation Unit, Biberach an der Riss, Germany

**Keywords:** biotherapeutics, polysorbate, tween, enzymatic degradation, HCP, mass spectrometry, fatty acids, particle formation

## Abstract

Polysorbates, in particular polysorbate (PS) 20 and 80, are the most commonly used surfactants for stabilising biotherapeutics produced by biotechnological processes. PSs are derived from ethoxylated sorbitan (a derivative of sorbitol) esterified with fatty acids of varying chain length and degree of saturation. In the past, these surfactants have been reported to have specific liabilities. Chemical (oxidations and hydrolyses) and enzymatic degradations have been reported to affect the stability of PS in drug products. Specifically, the presence of trace amounts (sub-ppm) of certain host cell proteins (HCPs) can induce enzymatic PS degradation, which can lead to the release of free fatty acids during storage over time. Enzymatic polysorbate degradation may impair the functionality of the surfactant in stabilising therapeutic proteins, leading to the formation of visible and/or sub-visible particles in biopharmaceutical drug products. This review summarises the enzymes currently known to be involved in the degradation of polysorbate in mammalian biotechnological processes for therapeutic proteins. In recent years, advanced analytical methods have been developed to qualify and quantify the PS-degrading enzymes. Most of these assays are based on mass spectrometry with a preceding HCP enrichment approach. Efforts were made to measure the enzyme activity and correlate it with observed PS degradation. The impact on drug product quality attributes, including fatty acid solubility and phase separation, up to the formation of visible particles, and the potential induction of protein and protein/fatty acid mixed particles as well as the sensitivity of specific PS quality towards enzymatic degradation, was considered. Various drug substance (DS) mitigation strategies related to the occurrence of PS degrading enzymes are discussed as amongst them the generation of stable HCP knockout cell lines, which are also carefully analysed. The underlying opinion article reflects the undergoing discussions related to PS degrading enzymes and focusses on (i) impact on drug product, (ii) analytics for identification/quantification (characterisation) of the PS degrading enzymes, (iii) enzyme activity (iv) currently identified enzymes, and (v) potential mitigation strategies to avoid enzymatic PS degradation during DS manufacturing.

## Introduction

**FIGURE 1:**
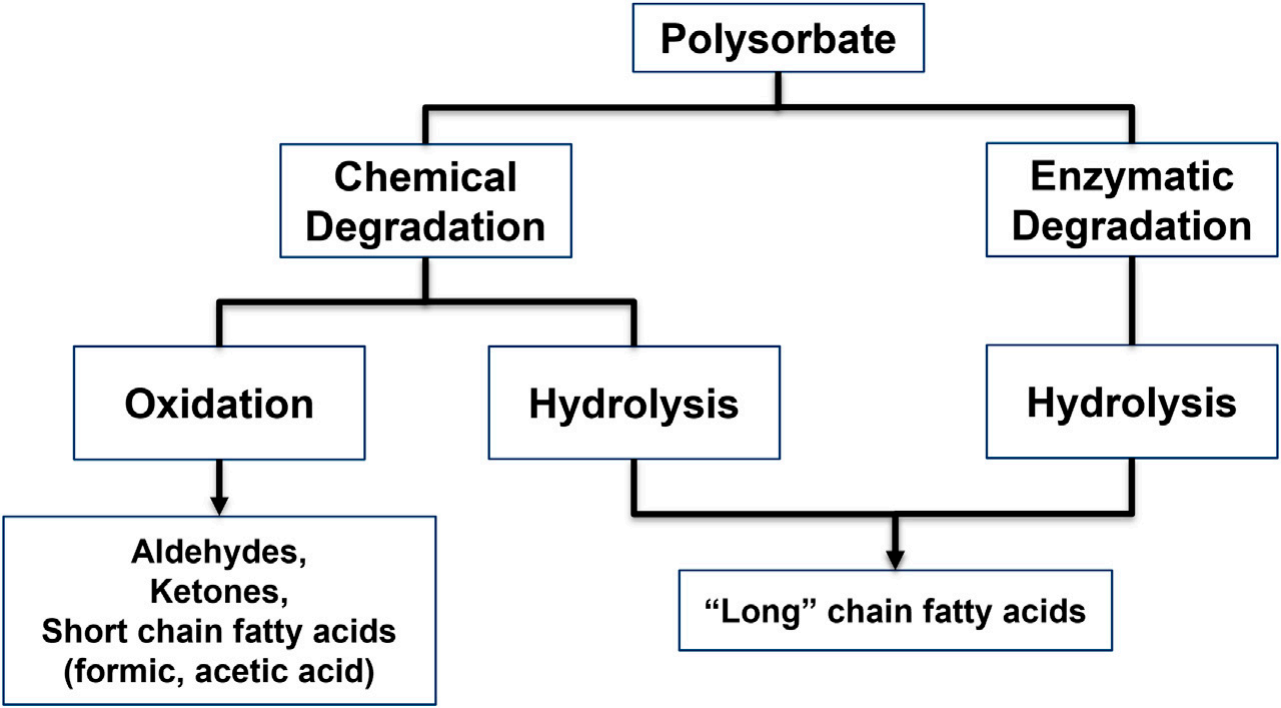
Polysorbate degradation pathways and the resulting main degradant classes.

Polysorbates, especially polysorbate 20 (PS20) and 80 (PS80), are the most predominantly used surfactants to stabilise biologics in drug product formulations (Strickley and Lambert, 2021; Wuchner et al., 2022a; Wuchner et al., 2022b). PS20 and PS80 are amphiphilic molecules which are derived from ethoxylated sorbitan (a derivative of sorbitol) esterified with fatty acids of varying chain length and saturation degree. Both PSs are composed of a large number of more than hundreds of single components (Evers et al., 2020). Previous studies have shown that polysorbates can degrade under certain conditions (Dwivedi et al., 2018; Ravuri, 2018). The article discusses two main degradation pathways: (a) chemical degradation with (a1) oxidation, (a2) hydrolysis and (b) enzymatic degradation (Figure 1) (Dwivedi et al., 2018; Ravuri, 2018). The review excludes further discussion of PS oxidation, as it has been recently covered in Kishore et al. (2011a), Kishore et al. (2011b), Kranz et al. (2019), Kranz et al. (2020), Mittag et al. (2022), Kovner et al. (2023), Kozuch et al. (2023), Weber et al. (2023), and Carle et al. (2024). Chemical hydrolysis under pharmaceutical relevant conditions, such as solution pH between 5 and 7, at 2°C–8°C is negligible (Dwivedi et al., 2020). However, since a few years, mounting evidence points towards a more prominent role of the enzymatic degradation of polysorbates as the major root cause of PS related particle formation in biopharmaceutical drug products (Tomlinson et al., 2015; Li et al., 2021; Li et al., 2022; Yuk et al., 2022; Zhang et al., 2022b).

The observed degradation dynamics of PS were found to be caused by tiny amounts of enzymes (HCPs), even at levels of parts per million (ppm) or below (Hall et al., 2016; Zhang et al., 2020b; Zhang et al., 2020c; Chen et al., 2021; Graf et al., 2021a; Graf et al., 2021b; Zhang et al., 2022b; Kovner et al., 2023). All of these reported enzymes share a common property: they induce the hydrolytic cleavage of the existing ester bonds in PS solutions, releasing free fatty acids from the polysorbate mixtures. In this case, the concentration of the released fatty acid molecules exceeds their corresponding solubility limits, fatty acid precipitation may occur, leading to the formation of sub-visible and/or visible particles (Kishore et al., 2011a; Doshi et al., 2015; Tomlinson et al., 2015; Doshi et al., 2020a; Glücklich et al., 2020). In other cases, due to the enzymatic polysorbate degradation and fatty acid release, the overall stabilising functionality of PS is impaired and consequently, the formation of protein particles may be formed as has been shown by Zhang et al. (2022a). Therefore, alternative surfactants have been discussed as both polysorbates are sensitive to hydrolytic ester cleavage caused by the presence of enzymatic host cell proteins (HCPs). However, this review will not consider this further as it has already been addressed elsewhere (Wu et al., 2021; Bollenbach et al., 2022; Ruiz et al., 2022; Li et al., 2024).

Until today, most of the known cases of HCP-mediated PS degradation were observed in biopharmaceutical manufacturing processes employing Chinese hamster ovary (CHO) derived production cell lines (Chiu et al., 2017; Valente et al., 2018; Chiu et al., 2017; Valente et al., 2015). The main reason why CHO cells have been most frequently reported in this conjunction may be because this cell type represents by far the most frequently used mammalian expression system for the industrial production of therapeutic glycoproteins (Kim et al., 2012).

Although there is limited literature on other mammalian cell lines, it is highly probable that the degradation of PS20 or PS80 by HCPs is not unique to CHO cells. This phenomenon is likely to be as critical in other expression systems, particularly those used for monoclonal antibody production, due to the native expression of lipases/hydrolases in almost all mammalian cell lines. It is currently unclear whether bacterial or fungal expression systems are also affected. However, cases similar to those found in CHO cells have not been reported thus far.

The present work summarises the current knowledge on enzyme-induced hydrolytic degradation of PS. Thereby, we focus on the following topics that are evaluated and critically discussed:

(i) enzymatic polysorbate degradation and its impact on drug product,
(ii) analytical tools used for the identification/quantification/characterisation of the enzymes
(iii) approaches to measure enzyme activity
(iv) a current list of identified enzymes
(v) potential mitigation strategies.

## Impact on drug product quality

Therapeutic proteins typically require a surfactant to maintain stability. During storage or when subjected to stress, protein particles of varying sizes and composition can form. Therefore, studies are performed to determine the minimal, functional surfactant concentration necessary to stabilise the corresponding proteins (Manning et al., 2010; Wagner et al., 2012; Ruiz et al., 2022; Zoeller et al., 2022). This has been described extensively in the last years (Carpenter et al., 2002; Bontempo, 2007; Shire, 2015; Kaur and Reusch, 2021). Since the appearance of enzymatic PS degradation in biologics, various studies have reported on the formation of FA containing particles, with the formation of “pure” FA particles, mixed FA-protein particles or protein particles (Kishore et al., 2011a; Tomlinson et al., 2015; Martos et al., 2017; Zhang et al., 2017; Roy et al., 2021).

### Fatty acid analytics and particle forensic

Several chromatographic methods have been presented to quantify and characterise fatty acids in drug product formulations (Ilko et al., 2015; Huang et al., 2018; Evers et al., 2021; Hoelterhoff et al., 2023) (Table 1). For the analysis of fatty acid particles, different extended characterisation techniques are used, such as, spectroscopic techniques, mainly mid-infrared or Raman spectroscopy to identify the presence of hydrocarbon chains in the particles (Garidel, 2002; 2013; Saggu et al., 2015; Dwivedi et al., 2018; Roy et al., 2021; Chen et al., 2023). Particle composition by mass spectrometry, electron microscopy and attempts to characterise particle morphology of sub-visible particles by flow imaging microscopy, backgrounded membrane imaging or total holographic characterisation have been presented (Saggu et al., 2021; Chen et al., 2023; Schleinzer et al., 2023).

**TABLE 1:** Most common analytical techniques used to investigate polysorbate degradation.

Analytical properties	Analytical approaches Examples	References
Polysorbate quantification	- Fluorescence micelle assay - Chromatography based as a single peak assay	Khossravi et al. (2002) and Lippold et al. (2017) Evers et al. (2020) Wuchner et al. (2022a) and Wuchner et al. (2022b) Kozuch et al. (2023)
Polysorbate degradation	Chromatographic assay coupled to different detectors - LC-CAD - LC-ELSD - LC-fluorescence - LC-MS - LC-UV	Li et al. (2014) Lippold et al. (2017) Evers et al. (2021), Webster et al. (2021), Wuchner et al. (2022a), and Wuchner et al. (2022b) Kozuch et al. (2023) Carle et al. (2024)
Particle formation	• Visual inspection according to the Pharmacopeia• Sub visible particles - Flow imaging microscopy - Backgrounded membrane imaging - Light obscuration	Siska et al. (2015) Saggu et al. (2021) Chen et al. (2023), Schleinzer et al. (2023), and Aryal et al. (2024)
Particle forensic	- Mid-infrared microscopy - Raman microscopy - Electron microscopy - EDX	Garidel (2002), Garidel (2013); Saggu et al. (2015), Dwivedi et al. (2018), and Hampl et al. (2018) Roy et al. (2021) Wuchner et al. (2022a) and Wuchner et al. (2022b) Chen et al. (2023)

CAD, charged aerosol detector; EDX, energy dispersive X-ray; ELSD, evaporative light scattering detector; LC, liquid chromatography; MS, mass spectrometry; UV, ultra violet.

### Formation of fatty acid particles

Siska and colleagues observed the formation of small clouds of particles in solutions when storing a developed monoclonal antibody drug in glass vials (Siska et al., 2015). The formulation contained PS20 as a surfactant. Particle analytics showed that the isolated particles consisted of free fatty acids. The distribution of the hydrocarbon chains found were consistent with those measured in the PS20 raw material. Particles, in a similar formulation, were also formed with PS80, but their formation was delayed compared to the use of PS20. The root cause of the presence of these particles showed that multiple lots of PSs, that were investigated for free fatty acid levels, exhibited differences based on polysorbate type and lot. Polysorbates purchased in more recent years exhibited greater distribution and quantity of free fatty acid, which increased the propensity to form particles.

Several cases, including the one described above, have been reported regarding the non-enzymatic formation of non-proteinaceous and/or fatty acid particles or the presence of higher levels of free fatty acids or impurities in the raw material (Siska et al., 2015; Hampl et al., 2018). Therefore, it is important to consider the quality control of polysorbate raw material, particularly regarding the presence of free fatty acids and “hydrophobic” impurities (Siska et al., 2015).

A clear observation related to the enzymatic degradation of PS in pharmaceutically relevant formulations, is an increase in the free fatty acid (FFA) content (Wuchner et al., 2022a; Wuchner et al., 2022b). In a recent industrial survey on the first indication of PS degradation in liquid products in vials, about 65% of the interviewed companies observed a decrease in PS content. Half of them experienced an increase in FFA levels, with the formation of subvisible (46%) and visible particles (38%) (Wuchner et al., 2022a).

Saggu et al. (2015) studied the composition of precipitated particles in a monoclonal antibody formulation that exhibited PS20 degradation using Raman spectroscopy. They found that the majority of the sub-visible particles identified were composed of mixtures of fatty acids. In a subsequent study, Saggu et al. (2021) identified particles in their drug product that did not exhibit signs of co-precipitation with protein. The flow microscopic investigation revealed that most particles had needle- and flake-shaped morphologies. This is consistent with previous findings (Cao et al., 2015; Saggu et al., 2015). The authors also underlined that subvisible particle counts in a relevant number (n = 11) of monoclonal antibody drug product stability batches did not correlate with the appearance of visible FFA particles. This was especially evident considering “freshly” manufactured drug product batches, where subvisible particle counts were very low at the beginning of the shelf-live period. As an explanation, Saggu and co-workers indicated that visible particles observed early on during stability are mostly composed of longer chain fatty acids with poor solubility based on their mass spectrometry data. They also noted the possibility of potential FFA nucleation by nucleation factors such as trace metal ions, which cannot be excluded (Doshi et al., 2015; Saggu et al., 2015; Allmendinger et al., 2021).

Yuk et al. investigated mitigation approaches for avoiding particle formation (FFA) induced by enzymatic hydrolysis of PS20 in protein-based drug products (Yuk et al., 2022). They conducted a full-factorial, design of experiment based longitudinal studies at 5°C and focused on three formulation parameters: (i) the concentration of monoclonal antibody in the drug product, (ii) the initial concentration of PS20 (HP quality, Croda), and (iii) the formulation pH. Based on the output of their study, two key formulation parameters, namely, protein and initial PS20 concentration had a considerable impact on particle formation and their onset. They concluded from their study investigating liquid formulated monoclonal antibody drug products in the presence of PS20, that “ (1) the shift to higher [mAb concentrations] is substantiated as a leading root cause for the increasing prevalence of FFA particle observations across the biopharmaceutical industry; (2) the risk of FFA particle formation is further exacerbated when the increase in [mAb concentration] is not counteracted by an increase in the initial [PS20 concentration] to enhance the FFA-solubilizing capacity of the formulation. (3) The effect of formulation pH in the 5–6 range is considerably less than the effect of [mAb concentration] or initial [PS20 concentration] or the interaction of [mAb concentration] and initial [PS20]” (Yuk et al., 2022). In summary, Yuk and colleagues proposed “to mitigate particle formation in DP formulated with PS20, the alternatives to consider—apart from shortening DP shelf-life or optimizing drug substance process to minimize levels of residual hydrolytic HCPs—are to decrease [mAb concentration] and/or increase initial [PS20 concentration]” (Yuk et al., 2022).

Although Yuk et al. (2022) were able to demonstrate a time-dependent onset of visible particles, which trended with the rise in subvisible particle counts and FFA levels and the decrease in PS20 concentration, this observation is different to the one by Saggu et al. (2021). Saggu et al. (2021) observed that subvisible particle counts in 11 mAb drug product batches on stability “did not correlate with the appearance of visible FFA particles in particular in young mAb drug product batches where subvisible particle counts were very low”. Such differences in the released fatty acids may be attributed to various factors, such as the enzymes promoting hydrolytic PS degradation and the solubility of the released fatty acid. The solubilisation property of the remaining PS micellar structure(s), also plays a role, highlighting the complexity of particle formation. Therefore, Yuk et al. (2022) noted that “the overall trends between SVPs and VPs observed here may not be generalizable” (Yuk et al., 2022). For more details, please refer to the studies of Saggu et al. (2021) and Yuk et al. (2022) as well as literature cited therein.

### Polysorbate quality and enzymatic degradation

The super refined (SR) polysorbate 20 quality from the supplier Croda was evaluated in a side-by-side comparison of PS20 HP versus PS20 SR for PS degradation, particle formation and protein stability (Doshi et al., 2020b). The PS stability comparison was performed under oxidative stress (with the addition of peroxides) and additionally in separate tests, in the presence of *Pseudomonas cepacea* lipase (PCL) and lipase B *Candida antarctica* (CALB) (both from Sigma Aldrich, St. Louis, MO). The enzymes CALB and PCL were chosen due to previous studies showing their opposing selectivity for PS20 component species. When using all-laureate PS20 (approximately 99% laurate fatty acid esters), McShan et al. (2016) found that enzymatic degradation in the presence of CALB resulted in a rapid decrease in monolaurate species, while displaying little activity on di- and tri-laurate species (McShan et al., 2016). PCL on the contrary had little hydrolytic specificity on mono-laurate species, while primarily degrading the higher order laurate species. As a result, a distinct enzymatic degradation profile and degradation products were produced.

Linked to enzymatic degradation, the results by Doshi et al. (2020b) suggest that PS20 SR is “less prone to particle formation than PS20 HP when there is preferential degradation of mono-esters of PS20, while more susceptible to particle formation when there is preferential degradation of higher order esters of PS20.” This observation has implications for evaluating and judging particle formation during enzymatic degradation.

For completeness, related to oxidative stress, PS20 SR showed higher levels of oxidative PS20 degradation, protein oxidation, higher peroxide generation rates and in some cases protein aggregation. To reduce the oxidative degradation, the authors proposed the use of methionine (10 mM) as an antioxidant (Doshi et al., 2020b). The authors have not identified a clear root cause for why PS20 SR is more susceptible to oxidative degradation than PS20 HP.

Overall, Doshi et al. (2020b) concluded with the “potential risks and benefits of PS20 SR compared to PS20 HP to enable a formulator to make an informed decision when choosing between the two surfactant grades in their drug product formulations.”

The impact of the fatty acid composition of PS80 seems to mirror the promotion of the formation of sub-visible particles. Pegues et al. (2021) investigated the effect of fatty acid composition in PS80 on the stability of therapeutic protein (rhG-CSF as filgrastim reference standard from USP and rituximab from Genentech) formulations. They used two types of PS80: (i) PS80 NF (from Spectrum Biochemical), which is a polysorbate synthesized from a fatty acid mixture containing mainly oleic acid (≥58%) and (ii) a PS80 (from Croda Inc., also denoted in other papers as AO-PS80) synthesized with high oleic acid (>98%). The stress conditions applied included high temperatures of up to 37°C and the addition of commercially available esterases, specifically Porcine liver esterase and mouse phospholipase B-like 2 (PLBD2). These stress conditions promoted the hydrolysis of the polysorbate ester bond and release of fatty acid. According to Pegues et al., the fatty acid composition of PS80 did not directly alter the stability profile of either therapeutic protein (measured by size exclusion chromatography), or significantly impact innate immune response or biological activity (rituximab Activity via antibody dependent cellular cytotoxicity assay) (Pegues et al., 2021). However, formulations containing PS80 NF exhibited a greater tendency to form sub-visible particles under stress conditions. This observation is discussed in the context of PS80 NF’s higher hydrocarbon chain heterogeneity, which is due to its composition of both saturated and unsaturated fatty acids, compared to the relatively pure monounsaturated oleic acid found in AO-PS80. Thus, Pegues et al. (2021) concluded that the initial composition of fatty acids in PS80 may promote sub-visible particulate formation under the tested stress conditions, but it may not impact protein aggregation or biological activity. The authors did not evaluate the fatty acid composition of the hydrolysate or the resulting insoluble fatty acid particles. They stated that additional studies would be beneficial to contribute to a better understanding of how purity of the fatty acid can be optimized to prevent particle formation in polysorbate-containing formulations.

In the same year, Doshi et al. published a report evaluating a modified version of High Purity PS20 (RO PS20 HP, Croda) (Doshi et al., 2021). The modified version contained lower levels of stearate, palmitate and myristate esters (Table 2) than the non-modified PS20 HP (Croda). The modification was designed to reduce the risk of FFA particle formation (Doshi et al., 2021). Using relevant stress approaches, the authors showed that interfacial protein protection and oxidation propensity were comparable between the two polysorbates indicating equivalent functionality between both PS20 qualities.

**TABLE 2:** Fatty acid ester distribution and specifications for RO PS20 HP and PS20 HP [according to Doshi et al. (2021), Table 3].

Fatty acid ester	RO PS20 HP /%	RO PS20 HP Specifications^a^ /%	PS20 HP /%	PS20 HP Specifications (USP^b^/EP^c^/ChP^d^) /%
C14:0 Myristic	15.9–16.9	14.1–18.1	18.4	14.0–25.0
C16:0 Palmitic	8.8–9.7	7.0–10.9	12.0	7.0–15.0
C18:0 Stearic	0.0–0.1	0.0–2.0	6.0	≤7.0 (EP) and ≤11.0 (USP/ChP)

^a^ RO PS20 HP, specifications fall within PS20 HP, compendial specifications.

^b^ United States Pharmacopeial Convention. United States Pharmacopeia and National Formulary. rockville, MD: United States Pharmacopeial Convention, 2018.

^c^ Council of Europe. European Pharmacopoeia. 9.3rd ed. Strasbourg, France: European Medicines Agency, 2017.

^d^ Chinese Pharmacopoeia Commission. Chinese Pharmacopoeia. 11th ed. Beijing, China: China Medical Science Press, 2020.

Enzymatic hydrolytic degradation, mediated by commercially available enzymes from Sigma Aldrich, such as *Mucor miehei* lipase MML, *C. antarctica* lipase CAL, *Pseudomonas cepacia* lipase PCL, *C. antarctica* lipase B CALB, delayed the onset of FFA particle formation in RO PS20 HP. The delay was more pronounced when higher order esters of PS20 were preferentially degraded. Furthermore, the hydrolytic degradants of RO PS20 HP formed fewer particles in the presence of spiked aluminium (Doshi et al., 2021). Doshi et al. (2021) “highlights the criticality of having tighter control on long [saturated] chain fatty acid ester levels of PS20 to reduce the occurrence of FFA particle formation upon hydrolytic degradation and lower the variability in its onset. By simultaneously meeting compendial PS20 specifications while narrowing the allowable range for each fatty acid ester and shifting its composition towards the shorter carbon chain species, RO PS20 HP provides a promising alternative to PS20 HP for biopharmaceutical DPs.” For transparency, one need to note that this study was co-authored by an employee of the polysorbate manufacturer (Doshi et al., 2021).

When comparing the composition difference of the three named fatty acid esters, stearate, palmitate and myristate esters, the main difference in composition between both PS20 quality grades is the “absence” of the C18:0 component in RO PS20 HP. The stearate component decreases by approximately 6%. In relation to the other two fatty acid esters, the reduction of myristate and palmitate is about 2%–3%. In summary, these fatty acid ester fractions are reduced to about 10%–12%. If this is relevant, according to the results provided by Doshi et al. (2021), one should consider in the future the fatty acid composition more carefully, especially the long chain, saturated fatty acid esters. Therefore, Doshi et al. (2021) emphasised “the importance of having tighter control on the FAE levels in the PS20 raw material to complement mitigation efforts against PS20 degradation by lowering the occurrence and reducing the variability of FFA particle formation.” This may delay the onset of particle formation.

### Fatty acid particle formation in the presence of specific impurities

As stated previously, in many cases, particle formation often occurs due to the accumulation of fatty acids released by the enzymatic hydrolysis of the polysorbate surfactant by co-purified host cell proteins. In addition, the presence of certain impurities may even amplify the formation of fatty acid particles. Gregoritza et al. (2022) reported on a metal-induced fatty acid particle formation which resulted from hydrolytic PS20 (High purity quality, from Croda, Edison, NJ, United States) degradation. They assessed the ability of various metal cations to cause the formation of fatty acid particles. It was found that the presence of trace amounts of multivalent cations (below 30 µM), particularly trivalent cations like aluminium (III) and iron (III), can act as nucleation seeds in the particle formation process. In the presence of divalent cations, such as calcium (II) or magnesium (II) the risk to induce particles is reduced. The phenomenon described can be explained by the ability of trivalent cationic metal impurities to form insoluble complexes with anionic FFAs due to their multiple opposing charges.

The authors emphasised that their observations resulted from studies where the presence of metal ions was studied independently. To mitigate the formation of metal-induced fatty acid particles, the authors propose testing chelators such as EDTA (EDTA ethylene-diamine-tetra acetic acid) and DTPA (DTPA diethylene-triamine-penta acetic) at least at a 1:1 M ratio, to reduce the risk of particle formation in biopharmaceutical formulations.

The observation that the presence of specific metal cations might be responsible for the nucleation of FFA particles in the presence of PS20 HP (Croda) was also reported by Allmendinger and co-workers (Allmendinger et al., 2021). Their data demonstrate the feasibility of nucleation of FFA particles in the presence of inorganic salts such as NaAlO_2_ and CaCl_2_ simulating relevant glass leachables and that the FFA particle formation depended on relevant aluminium concentrations. The concentration of the tested aluminium cation ranged from 0 to 0.250 μg/mL. Allmendinger et al. (2021) also investigated FFA particle formation in the presence of lauric/myristic acid and in the presence of different quantities and compositions of glass leachables. The glass leachables were obtained by several sterilization cycles using different types of glass vials. The formed particles were identified as a complex of glass leachables, including aluminium and FFAs, through mid-IR and SEM-EDX analysis. Based on this study, the author “highlight the complex interplay between (1) the presence of different FFAs in different concentrations; (2) the presence of different concentrations of intact PS and their degradation products (esters), potentially solubilising FFA particles; (3) the absolute concentration and combination of glass leachables; and (4) the kinetics and temperature dependence of particle formation. In particular, the relationship between (3) and (4) is currently unclear and warrants further investigation” (Allmendinger et al., 2021).

### Fatty acid particles and their fate during infusion

Doshi et al. (2021) have investigated the dissolution of fatty acid particles in drug products when diluted in intravenous infusion bags containing as dilution media such as 0.9% normal saline, 0.45% half normal saline or 5% dextrose. The study aimed to determine whether FFA particles in the DP dissolve in intravenous solutions prior to administration. Their assessment indicated that visible and/or sub-visible particles that contain high levels of lauric, myristic and palmitic acids dissolve immediately upon dilution (at or exceeding twofold) regardless of the intravenous bag or solution type. Therefore, the authors concluded that “the risk is low of visible and/or sub-visible particles, comprised of FFAs in biopharmaceutical DPs, being intravenously administered to a patient” (Doshi et al., 2021).

The fate of fatty acid particles in human plasma, especially its interaction with albumin has been considered within different studies (Doshi et al., 2021; Saggu et al., 2021; Schuster et al., 2022; Zhang et al., 2022a). Human serum albumin (HSA) is the primary macromolecular constituent of serum with concentrations of up to 50 mg/mL. Kim et al. (2022) demonstrated that the presence of HSA, even at concentrations well below the physiological range (tested below 10 mg/mL HSA), can reduce the formation of fatty acid particles in polysorbate-containing solutions. They reported that the presence of HSA is sufficient to prevent the formation of fatty acid particles. Furthermore, they demonstrated that HSA rapidly and completely solubilize pre-formed particles. The rational for this observation is due to the primary biological function of HSA as a metabolite transporter. HSA shows a total of nine binding sites for fatty acids (Krenzel et al., 2013). Based on this, Kim et al. (2022) hypothesized that HSA may prevent and potentially reverse particle formation by directly binding to fatty acids released by the action of host cell lipases (Kim et al., 2022; Schuster et al., 2022). Besides the binding of free fatty acids to albumin, calorimetric binding studies have shown, that PS also binds to albumin (Garidel et al., 2009). These results provide a plausible mechanistic explanation for previous observations that the presence of albumin is able to “dissolve” fatty acid particles and diminishes concerns regarding low levels of particles in the final DP formulations (Kim et al., 2022). In this context, the particles need to be characterised carefully.

Saggu et al. (2021) were also interested in this topic and investigated the effects of exposing visible FFA particles to saline and human plasma to shed light on the dilution and/or administration, a process that is routinely performed in the clinical setting. In the described experimental approach, no precipitation of human plasma proteins or particle growth was observed (Saggu et al., 2021). They concluded that there was little to no effect of human plasma exposure on their monoclonal antibody DP, and the presence of FFA particles. However, the authors mentioned that “the presence of visible product-related particles during stability storage in any drug product requires a thorough product-specific safety assessment that, at minimum factors in the mechanism of action of the drug, the route of administration, patient population as well as the visible particle size/count. In the case of fatty acid-related particles, exposure to soluble fatty acids should be evaluated in the context of patient safety. In addition, implementation of in-line filters needs to be considered. Additional studies, such as dissolution studies or human plasma exposure can support the safety assessment, e.g., the risk for occlusion of blood vessels is lower if it was demonstrated that particles re-dissolve” (Saggu et al., 2021).

## Enzymatic degradation: polysorbate preference

When considering the impact of enzymatic degradation on the cleavage of the ester bond of polysorbate, it is important to take into account the following aspects: (i) identification of the enzyme and determination of its enzymatic activity, (ii) preference for a specific type of polysorbate (PS20 vs. PS80) and (iii) the quality of polysorbate (HP, MC, AO, AL).

A few years ago, Hall et al. (2016) reported on the enzymatic degradation of PS20 and PS80 in products derived from a CHO-based biologics manufacturing process. To identify the enzyme(s) involved, they enriched the enzymatic activity from CHO cell culture supernatant and subjected the isolated proteins to a shotgun proteomic approach. This approach identified the presence of group XV phospholipase A2 isomer X1 (LPLA2). LPLA2 is an enzyme that was shown to degrade lysophosphatidylcholine to glycerophosphoylcholine releasing a fatty acid. The material for formulation experiments was produced using recombinant LPLA2 (r-LPLA2), which was overexpressed in CHO cells and purified to confirm its functional integrity (Hall et al., 2016). They were able to show that LPLA2 hydrolysed PS20 and PS80 (both from T.J. Baker Phillipsburg, NJ, quality not further specified) in a concentration and time-dependent manner. Furthermore, Hall et al. (2016) identified endogenous LPLA2 in three purified monoclonal antibody products at concentrations less than 1 ppm. They also demonstrated PS degradation in these three mAb products. In contrast, an antibody product without detectable amounts of LPLA2 did not show significant PS hydrolysis (Hall et al., 2016).

Hall et al. (2016) also noted that the rates of PS hydrolysis among the different antibody samples they have tested, “were not compared as subtle differences, such as mAb concentration and polysorbate content, in their formulations may impact polysorbate degradation.” In their study comparing the enzymatic sensitivity of PS20 and PS80, Hall et al. (2016) concluded that PS20 exhibited a different hydrolysis profile in the presence of rLPLA2 compared to PS80. The tentative explanation is because the higher-order esters of PS20 are not as stable as ones of PS80 against rLPLA2 and hydrolysis of higher-order esters generates lower-order esters, that is, diester to monoester, triester to diester, and so forth. Thus, PS20 monoester intensity will not significantly change before the higher-order esters are completely hydrolysed. On the other hand, the higher order esters of PS80 are much more resistant against rLPLA2 compared to monoesters, that is, their hydrolysis with rLPLA2 is much slower than the monoester. Thus, loss of monoester was complete before a significant change in the intensity of the higher-order esters was observed (Hall et al., 2016). Even at low concentrations of rLPLA2 of 0.1 ppm, an incubation for 5 days at 37°C resulted in a 30% loss of PS80 and a 10%–15% loss of PS20 (Hall et al., 2016). The differences described in the hydrolysis of PS20 and PS80 were discussed by Hall et al. (2016) in the context that the head groups of PS20 are similar to those of PS80 but changes in the hydrocarbon chain composition were evident. The main components of PS20 are laurate whereas oleate is the predominate component of PS80. The tail groups (fatty acids) are more hydrophobic in PS80 than in PS20 and higher order esters are more hydrophobic than monoesters. Therefore, the critical micelle concentration range of PS80 is lower than that of PS20 and of higher order esters than that of monoesters (Knoch et al., 2021). “RLPLA2 is likely to prefer the free form of polysorbates in solution, and polysorbates in the micelle state should resist rLPLA2. This explains why hydrolysis of PS80 monoester is much faster than that of higher-order esters. For PS20, the critical micelle concentration of higher-order esters is much higher than that of PS80; therefore, the hydrolysis rate of higher-order PS20 with rLPLA2 is greater. When the higher order ester is hydrolysed, it forms one less order ester” (Hall et al., 2016).

To confirm these results, the authors proposed to engineer the CHO host cell line to eliminate the endogenous expression of LPLA2 and to repeat the formulation experiments to determine whether they are stabilised, i.e., reduced polysorbate hydrolysis, using the LPLA2 knockout approach (Hall et al., 2016). In conclusion, the study by Hall et al. (2016) identified a specific enzyme, LPLA2, which is responsible for PS degradation, but they cautioned us that their study does not exclude the potential involvement of other enzymatic activities, as they identified additional enzymes at trace levels, and it would need to be determined whether these unknown enzymes may also have the potential to degrade polysorbates.

In this context, Glücklich et al. (2021) have used three different surrogate enzymes [lipoprotein lipase from *Burkholderia* sp. (LPL), lipase from porcine pancreas Type II (PPL F2) and Type VI-S (PPLF6)] with distinctly different degradation kinetics and different degradation fingerprints with respect to the hydrolysis of the mono- and multi-esters of PS20 HP and PS80 HP. The observed degradation preferences of the surrogate lipases regarding PS substrate are: (i) LPL–degrades mainly di- and multi-esters; (ii) PPL F2 – degrades mainly mono-esters; (iii) PPL F6 – degrades all ester types “evenly” (Glücklich et al., 2021).

Using an activity-based protein profiling (ABPP) approach, Li et al. (2021) isolated the serine hydrolase phospholipase A2 group VII (PLA2G7/PAF-AH) and showed that it contributes to the degradation of PS80 (quality not further defined). In the same year, Zhang and colleagues used an enrichment approach for HCPs by immunoprecipitation followed by shotgun proteomics to identify the HCP sialate O-acetylesterase (SIAE), which showed strong enzymatic activity towards PS20 SR (SR super refined quality from Croda) degradation even at low concentrations (<5 ppm level) (Zhang et al., 2021). They incubated recombinant SIAE with PS20 SR and detected a unique degradation pattern in which the hydrolysis of monoesters with short fatty acid chains (C12, C14) was observed, but not that of monoesters with long fatty acid chains (C16, C18) or higher-order esters (Zhang et al., 2021). They detected and quantified SIAE in several formulated mAbs. The amount of SIAE was positively correlated with the degradation of PS20 SR in these mAbs during incubation. Additional experiments by the authors showed that when SIAE was depleted using a Dynabeads Antibody Coupling Kit, PS20 SR degradation was reduced, suggesting a causal relationship between SIAE and PS20 degradation (Zhang et al., 2021). The lipase activity of SIAE appeared to be specific for PS20 SR, but not for PS80 SR, which contains monoesters with long chain fatty acid (C18) and higher order esters. The polysorbates tested by Zhang et al. (2021) were of super-refined quality grade (Croda, East Yorkshire, United Kingdom) and whether the observed results can be confirmed for multi-compendial polysorbates, needs to be clarified.

A study by McShan et al. (2016) focussed on investigating the hydrolytic degradation of PS20 and PS80 due to the presence of specific carboxylester hydrolases. Although, the enzymes used were not present/expressed by CHO cells and therefore not directly reflecting “real-life processes,” this approach is relevant for unravelling enzyme-specific preferences for PSs (McShan et al., 2016). The carboxylester hydrolases tested by McShan et al. (2016) included those from *Pseudomonas cepacian (PCL)*, *Thermomyces lanuginosus (TLL)*, *C. antarctica lipase B (CALB)*, rabbit liver esterase (RLE), and pig pancreas lipase type II (PPL). The surfactants investigated were PS20, containing approx. 99% laurate fatty acid esters, and which were synthesized by BASF SE (Ludwigshafen, Germany) and PS80, which contained approx. 98% oleate fatty acid esters, HX2 Ultra-Purity grade, which were synthesized by NOF (Irvine, CA) (McShan et al., 2016). From their investigation, McShan et al. (2016) concluded that key PS components are uniquely hydrolysed by different carboxylester hydrolases in a specific pattern, resulting in enzyme-specific PS degradation profiles. For specific enzymes, PCL and TLL, PCL was shown to be more active against PS20 than PS80 components, except for POE sorbitan monoester. CALB is active against PS80, particularly POE sorbitan monooleate. In agreement with the results of Plou et al. (1998), RLE and PPL were shown to be active against PS80. The question remains as to why some enzymes have higher hydrolytic activity towards certain PS components, although the reaction is likely to be governed by the availability of different enzyme active sites to accommodate bulky hydrophobic POE or ester moieties (Plou et al., 1998; McShan et al., 2016; Glücklich et al., 2021). McShan et al. (2016) highlighted that “none of the PS components in either PS20 or PS80 were completely resistant to hydrolysis by all of the enzymes tested. Although the data do tentatively suggest a “possible” advantage of PS80 versus PS20 in terms of the reduced rate of the tested enzyme-mediated hydrolysis for the specific PS components, such as di- and trioleate esters, PS80 has been shown to be more prone to oxidation than PS20” (Borisov et al., 2015; Kozuch et al., 2023; Weber et al., 2023; Borisov et al., 2015; Kozuch et al., 2023; Weber et al., 2023).

In a recent report by Kovner et al. (2023), the authors tested 20 enzymes that they had previously identified as having hydrolytic activity against PS [see Table 3 in Kovner et al. (2023)]. Of these 20 enzymes, they identified 13 enzymes to have PS20 degrading activity including novel enzymes, which were not previously described to degrade PS. The enzymes were recombinantly expressed in CHO cells, purified, and characterized using selected methods (Graf et al., 2021b). Hydrolytic activity was assessed using a fluorogenic esterase substrate assay with MU-C8 (4-methylumbelliferyl caprylate) as substrate (Bhargava et al., 2021). Of the 20 recombinant enzymes tested, 6 showed no activity against the MU-C8 substrate in the esterase activity assay. The enzyme concentration generally tested ranged from 50 to 0.05 μg/mL. In addition, these enzymes were tested with regards to their activity against PS20 (High Purity grade from JT Baker, Radnor, PA) at 0.2 and/or 0.4 mg/mL in representative protein formulations. Kovner and co-workers demonstrated an expected difference in results for some lipases/hydrolases due to the differences in substrate (MU-C8 versus PS20) (Kovner et al., 2023). The authors also investigated the enzyme activity in the presence/absence of a mAb and showed that in some cases (e.g., for rhLPL, LAL and PPT1) the presence of the antibody decreased the enzyme activity during their screening experiments.

**TABLE 3:** Hydrocarbon chain composition of polysorbate 20 and 80 according to the European pharmacopoeia (Ph. Eur), United States Pharmacopoeia convention (USP) and the Japanese Pharmacopoeia (JP).

Fatty acid, chemical formula	Ph. Eur		USP		JP	
	PS20	PS80	PS20	PS80	PS20	PS80
Caproic, C_6_H_12_O_2_ /%	≤1		≤1			
Caprylic, C_8_H_16_O_2_ /%	≤10		≤10			
Capric, C_10_H_20_O_2_ /%	≤10		≤10			
Lauric, C_12_H_24_O_2_ /%	40–60		40–60			
Myristic, C_14_H_28_O_2_ /%	14–25	≤5	14–25	≤5	—	≤5
Palmitic, C_16_H_32_O_2_ /%	7–15	≤16	7–15	≤16		
Stearic, C_18_H_36_O_2_ /%	≤7	≤6	≤7	≤6	—	≤6
Oleic, C_18_H_34_O_2_ /%	≤11	≥58	≤11	≥58	—	≥58
Linoleic, C_18_H_32_O_2_ /%	≤3	≤18	≤3	≤18	—	≤18
Linolenic, C_18_H_30_O_2_ /%	—	≤4	—	≤4	—	≤4

**FIGURE 2:**
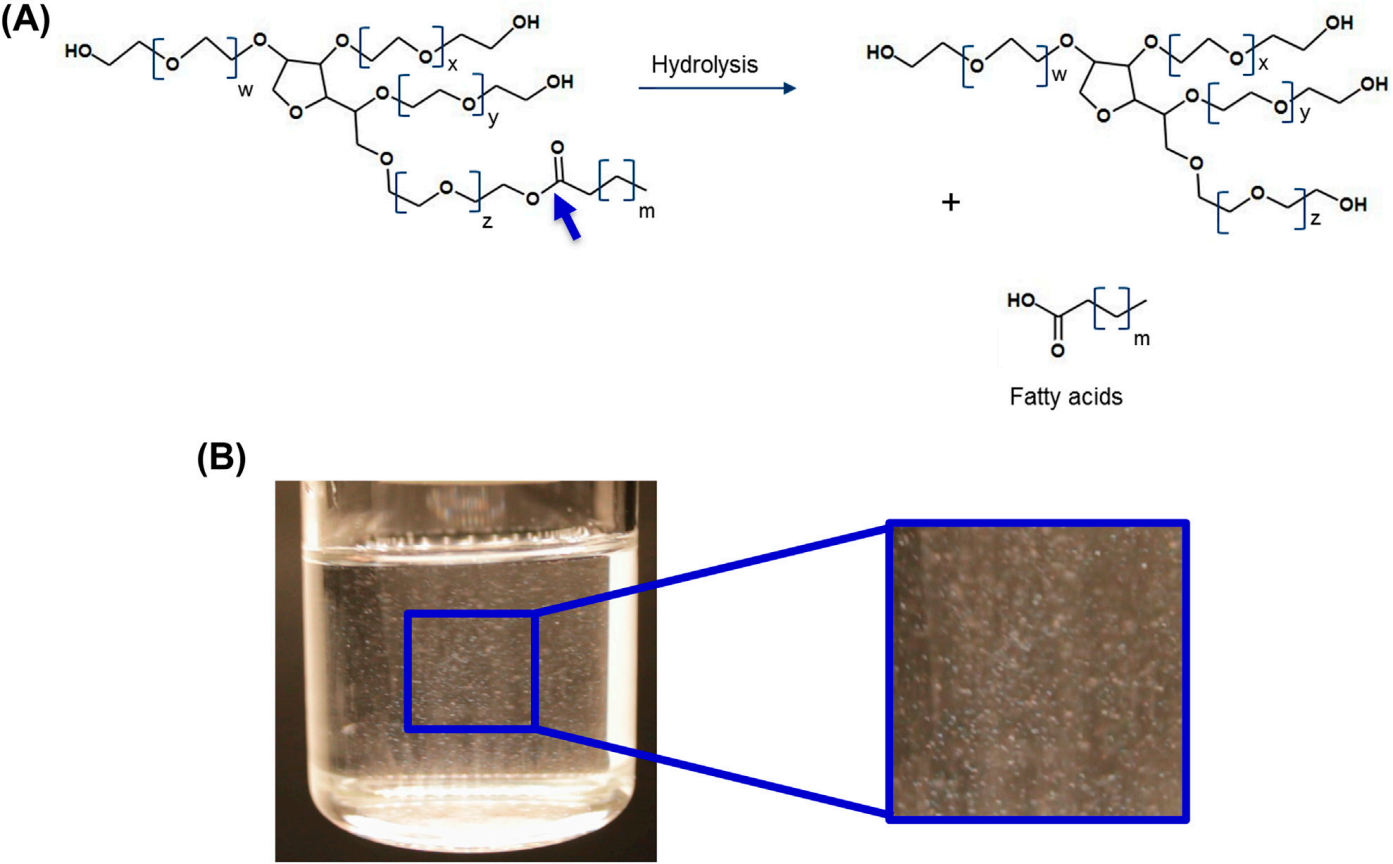
**(A)** Polysorbate degradation due to enzymatic cleavage of the ester bond and the release of fatty acids. **(B)** Formation of fatty acid particles. The resulting particles may vary in size from sub-visible to visible. The particles shown are between 100 and 400 μm in size. The size of the fatty acid particles is dependent on the amount of free fatty acid present, solubility limit, the chemical nature of the fatty acid hydrocarbon chain, as well as on the solution properties (e.g., temperature or presence of specific excipients) and protonation of the fatty acids (Dwivedi et al., 2018) W + X + Y + Z = 20.

Eight enzymes (PPT1, LAL, rhLPL, LPLA2 CES1F, CES1, SMPD1 and SIAE) were evaluated for their ability to degrade PS80 (Ultra pure HX2, all oleate grade from NOF America, San Mateo, CA) using a chromatographic content assay and their conclusion was: “These results found that of the enzymes tested, the ability to degrade PS80 was similar to PS20” (Kovner et al., 2023). A few selected enzymes (rhLPL, LAL, PPT1) were more deeply investigated to evaluate enzymatic activity differences and substrate specificity between both polysorbates. In this context, enzyme kinetics studies were performed. The kinetic profiles of the three tested enzymes (rhLPL, LAL, PPT1) against PS20 and PS80 differed leading to the conclusion that the presented “results provide further evidence that PS80 tends to be less susceptible than PS20 to hydrolysis by the recombinant enzymes tested here” (Kovner et al., 2023). On the other hand, according to the data provided by the authors, the decrease in intact PS20 is faster for certain enzymes compared to PS80, at a first sight, the decrease in PS20 content follows an asymptotic course, so that after a certain time a plateau is reached. Regarding the decrease of intact PS80, a linear decrease is however more “likely” according to the published data [see Figure 2 in Kovner et al. (2023)]. When comparing the remaining intact PS content after 14 h in the experiments presented by Kovner et al. (2023) (compare Figure 2 in their paper), the differences between PS20 and PS80 degradation were less obvious and extrapolation to, e.g., 30 h could change the statement mentioned above. It is therefore very difficult to show whether the enzymes have a specificity for PS20 or PS80. More studies, especially long-term, real-time studies monitoring PS degradation directly would be more informative.

The data presented by Kovner et al. (2023) supported observations, which were previously described by others, that different PS-degrading enzymes have a distinct specificity towards the different esters in the PS blend, potentially allowing enzymes to be categorised according to their “enzyme fingerprints” (McShan et al., 2016; Glücklich et al., 2021; Zhang et al., 2022b; Kovner et al., 2023). This could have an impact on the stabilising properties of PS depending on which PS ester fraction is more susceptible to hydrolysis (Tomlinson et al., 2020; Diederichs et al., 2023; Gregoritza et al., 2024).

In a recent study, Gregoritza et al. (2024) investigated the enzymatic susceptibility of PS20 (SR quality from Croda) and concluded that the stability of liquid biopharmaceutical formulations is dependent on the degradation pattern of PS20. Essentially, degradation of PS20 HOE, as observed for MML, results in a higher risk of FFA particle formation, whereas depletion of PS20 monoesters increases the risk of protein particle formation. As a result, the different enzymes or mixtures of enzymes that may be present in drug substance and drug product have a significant impact on stability. They suggest that the effect of the enzymatic PS20 degradation profile on stability should be systematically investigated using a larger number of mAbs of different formats (Gregoritza et al., 2024). This issue of variable PS degradation patterns and the resulting different compositions of the formed (mix)-micelles also has an impact on the content method based on the micelle-based assay (Martos et al., 2020; Glücklich et al., 2021). Some examples are summarised in Table 4.

**TABLE 4:** Specificity of selected PS degrading enzymes towards the various esters in the PS mixture (Kovner et al., 2023).

Enzyme	Observations
PPT1	Strong preference for largely degrading more hydrophobic mono-esters and higher order esters, while leaving some of the most hydrophilic mono-esters intact
rhLPL	Degrade all esters concurrently
LAL	Degrade all esters except the sorbitan-POE monolaurate peak, which is left largely intact
HACH	Strong preferences for the mono-esters of PS20
CES1F	Strong preferences for the mono-esters of PS20
LPLA2	Preference for esters in the middle range of hydrophobicity, largely leaving both the most hydrophilic and most hydrophobic components intact
SMPD1	A weakly active enzyme, preferentially degrades higher order esters

This shows that enzymes have a different enzymatic hydrolysis “fingerprint” when degrading polysorbates. The reason for this is not straightforward. PS20 and PS80, as surfactants, form micellar structures above their critical micelle concentration range (CMCR) of sizes between 6 and 10 nm in diameter, depending on solution conditions, temperature, etc (Garidel et al., 2017; Garidel et al., 2021). The CMCR for PS80 is lower compared to PS20 (7–16 µM vs. 15–75 µM based on surface tension measurements). Knoch et al. (2021) showed that the PS have much more complex micellisation properties than usually assumed according to their calorimetric approaches. Above their CMCR, the micellar structures coexist with monomeric polysorbate molecules. It is discussed that the enzyme somehow interacts with the monomeric PS molecule and catalyses the hydrolysis of the fatty acid ester bond. Thus, the association of PS molecules in micellar structure(s) may provide a degree of “self-protection” for the PS molecules, creating hydrophobic exclusion surfaces that may inhibit hydrolase activity.

## Methods for the identification, characterisation and quantification of polysorbate degrading enzymes

**FIGURE 3:**
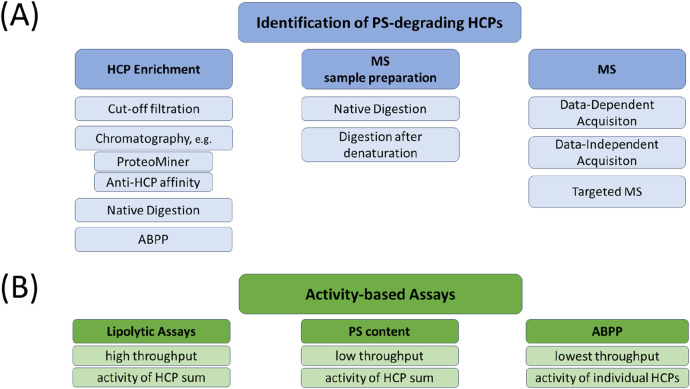
Overview of analytical tools used for the identification, characterisation, and quantification of PS-degrading HCPs. **(A)** Identification of low abundant HCPs is based on three different steps: HCP enrichment, sample preparation, and mass spectrometric analysis. Different technologies for each step are depicted and can also be combined in order to increase the sensitivity for HCP identification. Depending on the experimental set-up, HCPs of interest can also be (relatively) quantified within the mass spectrometric analysis. **(B)** In order to determine the overall activity of PS degradation within samples of interest, a lipolytic assay can be applied also offering the possibility for high throughput analysis. Alternatively, activity towards PS degradation of a sample can also be measured by methods determining the PS content (see Table 1) comparing differently stressed samples. In contrast to the methods mentioned so far, ABPP offers the possibility to evaluate the PS degradation activity of each individual HCP, which comes along with a lower throughput. *For references see text*. ABPP, Activity-Based Protein Profiling; HCP, Host Cell Protein; MS, mass spectrometry; PS, polysorbate.

Li et al. (2022) provided an overview of the analytical toolbox used to control PS degradation. Due to the low abundance of HCPs compared to the high proportion of biopharmaceutical products, specific requirements such as HCP enrichment and product depletion are necessary for assays to identify traces of enzymes responsible for PS degradation, even when using advanced instrumentation and methods such as mass spectrometry-based proteomics (Guo et al., 2023). Published examples of promising methods for the investigation of low abundance HCPs are provided below. Figure 3 provides an overview of the analytical toolbox. Table 5 summarises selected, currently known polysorbate degrating enzymes according to publications of Kovner et al. (2023), Li et al. (2021), Dehghani et al. (2023) and Liu et al. (2022).

**TABLE 5:** Summary of main currently known polysorbate degrading enzymes.

Hydrolase	Gene ID	PS degrading activity based on Kovner et al. (2023)	Li et al. (2021)	Dehghani et al. (2023)	Liu et al. (2022)	Synonym	Uniprot Acc. No.	References
Carboxylic ester hydrolase	CES-2c	YES	NO	YES	YES	Acylcarnitine hydrolase (Liver carboxylesterase)	G3IIG1	Li et al. (2021), Liu et al. (2022), Dehghani et al. (2023), and Kovner et al. (2023)
Carboxylesterase 1	CES-1	NO	YES	YES	NO	Live carboxylestherase 1-like protein	A0A061IFE2/A0A061ID92	Zhang et al. (2020b) and Gupta et al. (2023)
Carboxylesterase 1f	CES1F	YES	NO	YES	NO		A0A8C2MA83	90% ID with CES-B1L, (Gupta et al., 2023)
Liver carboxylesterase B-1-like protein	CES-B1L	NOT INCLUDED	NO	NO	NO		A0A061I7X9/A0A061IAA7	Zhang et al. (2020c)
Isoamyl acetate hydrolyzing esterase 1 (putative)	IAH1	YES	YES	YES	YES		G3IHH9	Liu et al. (2022) and Kovner et al. (2023)
Lipase A	LAL | LIPA	YES	NO	YES	YES	Lysosomal acid lipase	G3HQY6	Kreimer et al. (2017), Graf et al. (2021b), Yang et al. (2021), Liu et al. (2022), Zhang et al. (2022b), 2023, Gupta et al. (2023), and Kovner et al. (2023)
Lipoprotein lipase	rhLPL	YES	YES	YES	YES		Q6IAV0 (human) G3H6V7 (CHO)	Levy et al. (2016), Chiu et al. (2017), MacDonald et al. (2018), Graf et al. (2021b), Li et al. (2021), Yang et al. (2021), Liu et al. (2022), Zhang et al. (2022b), and 2023; Gupta et al. (2023)
Lysophospholipase-like protein 1	LYPLAL1	YES	YES	YES	YES	Palmitoyl-protein hydrolase	A0A8C2QJB4 G3GRE5	Bürger et al. (2012), Li et al. (2021), Liu et al. (2022), and Gupta et al. (2023)
Lysophospholipase-like protein 2	LYPLA2	NOT INCLUDED	YES	YES	YES	Acyl-protein thioesterase 2/palmitoyl-protein hydrolase	G3IP80	Bürger et al. (2012), Li et al. (2021), Liu et al. (2022), and Gupta et al. (2023)
N-acylsphingosine amidohydrolase 1	ASAH1	NO	NO	YES	NO	Acid ceramidase	G3GZB2	Graf et al. (2021a), Li et al. (2021), Yang et al. (2021), and Zhang et al. (2023)
Peroxiredoxin 1	PRDRX1	NOT INCLUDED	NO	NO	NO		Q9JKY1	Liu et al. (2019) and Singh et al. (2020)
Peroxiredoxin 6	PRDRX6	NO	NO	NO	NO		A0A8C2LBB3 A0A8C2L953	Uniprot accession number from publication is obsolete (A0A3L7HKA7) Fisher (2017)
Palmitoyl-protein thioesterase 1	PPT1	YES	NO	YES	YES		G3HN89	Graf et al. (2021b), Yang et al. (2021), Hecht et al. (2022), Liu et al. (2022), Gupta et al. (2023), Kovner et al. (2023), and Zhang et al. (2023)
Palmitoyl-protein thioesterase 2	PPT2	NOT INCLUDED	YES	YES	NO	Lysosomal thioesterase	G3HZC7	Li et al. (2021)
Phospholipase A1 member A	PLA1A | PLALA	YES	NO	YES	NO		G3I1J5	MacDonald et al. (2018), Li et al. (2021), and Kovner et al. (2023)
Platelet-activating factor acetylhydrolase	PLA2g7 | Paf-Ah	NOT INCLUDED	YES	YES	YES		Q13093 (human) G3I3E7 (CHO)	E. et al., 2023 (no pblast hit in CHO) Liu et al. (2022) and Gupta et al. (2023)
Phospholipase D family member 3	PLD3	NO	NO	NO	NO		G3HNQ5	Yang et al. (2021) and Kovner et al. (2023)
Phospholipase A2 group XV	LPLA2 | Pla2g15	YES	YES	YES	YES	Lysosomal phospholipase A2	G3HKV9	Shayman et al. (2011), Hall et al. (2016), McShan et al. (2016), MacDonald et al. (2018), Li et al. (2021), Yang et al. (2021), Liu et al. (2022), and Gupta et al. (2023)
Protein phosphatase methylesterase 1	PPME1	NOT INCLUDED	YES	NO	NO		A0A9J7JJI7	
Sialic acid acetylesterase	SIAE	YES	YES	YES	NO		G3IIB1	Li et al. (2021), Zhang et al. (2021), and Gupta et al. (2023)
Sphingomyelin phosphodiesterase 1	SMPD1	YES	NO	YES	NO		G3IMH4	Yang et al. (2021) and Gupta et al. (2023)
Tyrosyl-DNA phosphodiesterase 1	TDP1	YES	NO	NO	NO		G3HBG4	No literature found

The core of the list is based on two approaches, one is the systematic recombinant expression and characterisation (Kovner et al., 2023), the other one is the activity-based protein profiling (Zhang et al., 2020c; Li et al., 2021; Liu et al., 2022; Dehghani et al., 2023). Please note that the list does not aim to collect all the enzymes, rather focus on critical and well characterized HCPs and list some examples where the complexity of assessing the activity and effect of these HCPs is well presented.

### Cut-off filtration

A simple and powerful strategy to identify media-abundant HCPs in antibody drugs using a single-step of molecular weight cut-off filtration of 50 kD followed by shotgun proteomics analysis is described by Chen et al. (2020b). This method is capable of detecting levels of spiked HCPs at concentrations as low as 1 ppm. 150 HCPs were detected by analyzing a NIST (National Institute of Standards and Technology) mAb formulation. We believe that this method lacks sensitivity to detect very low levels of polysorbate degrading enzymes especially as some polysorbate degrading HCPs such as Lysosomal acidic lipase (LAL, 45 kDa), Phospholipase A2 (PLA2, 14–18 kDa) and Phospholipase c (PLC, 35–50 kDa) have molecular weight ranges of below 50 kDa. In agreement with the authors, we suggest that further additional and complementary methods to detect lower levels of HCPs are required for comprehensive HCP investigations.

### ProteoMiner

The ProteoMiner technology was introduced by Boschetti and Righetti (2008) to enrich low to medium abundance proteins while reducing the levels of high abundance proteins. It is based on a combinatorial ligand library made of millions of hexapeptides immobilised on beads (Boschetti and Righetti, 2008). Proteins and protein complexes will bind to specific peptide ligands mainly through hydrophobic interactions or other weak interactions including ionic interactions and hydrogen bonding. High abundance HCPs and the biopharmaceutical will easily saturate their limited number of ligands and therefore be washed out. Low abundance proteins do not saturate their hexapeptide ligands and are therefore enriched. Using this technology Chen et al. (2020c) tripled the number of HCPs identified in the above mentioned NIST mAb formulation (500 HCPs were confidently identified). With parallel reaction monitoring (PRM) results, they confirmed that the novel HCPs found by this method were enriched between 100- and 400-fold. Two years later, the same lab published another study in which they were able to increase the sensitivity of this method by coupling the ProteoMiner technology with limited digestion (Zhang et al., 2022b). Low abundance HCPs were enriched up to 7694-fold and levels of 2 ppb were detectable. 850 HCPs were detected in the NIST mAb preparation, which is 40% more than with ProteoMiner alone. Details of the native digestion are described separately below. We believe that by detecting HCPs at the ppb level, there is a fair possibility of identifying enzymatic HCPs responsible for polysorbate degradation, which can be active on PS degradation over a period of days or weeks.

### Anti-HCP affinity chromatography

The following two publications describe the use of anti-HCP affinity chromatography to reveal the antibody coverage for characterising the anti-HCP antibody coverage of an ELISA antibody reagent. Using anti-HCP affinity chromatography in a 96-well ELISA-plate formate, Pilely et al. (2020) identified approximately 1'000 different HCPs in early process steps of *E. coli* products for each of 3 commercially available anti-E. coli HCP-ELISA antibody reagents in *E. coli* derived products by subsequent MS preparation and analysis. Waldera-Lupa et al. (2021) used anti-HCP affinity chromatography in a bead-based format and identified approximately 150 HCPs in the highly purified downstream UF/DF step, the process prior to the final formulated Bulk Drug Substance (BDS). Although the focus of these publications was on the characterisation of the anti-HCP antibody reagent, the sensitivity of the anti-HCP affinity chromatography to detect small traces of HCP became apparent. However, there are still limitations to this method, including the fact that the number of HCPs identified in BDS is highly dependent on the MS evaluation criteria chosen, e.g., Amanda score, number of unique peptides and other parameters. Although the antibody coverage may bias the results towards immunogenic HCPs as these HCPs are expected to induce antibodies in the animal and thus bind to the HCP in anti-HCP affinity chromatography. The different levels and variable affinity of anti-HCP antibodies also make it difficult to quantify individual HCPs in BDS after the anti-HCP affinity chromatography step.

### Protein A and anti-HCP affinity chromatography

Graf et al. (2021b) developed a comprehensive enrichment approach for HCPs, employing both Protein A and anti-HCP affinity chromatography. This approach enabled a thorough analysis of the HCP population in an antibody formulation that is susceptible to PS hydrolysis. Liquid chromatography coupled with tandem mass spectrometry was used to identify HCPs. Several enzymes classified as hydrolases were then recombinantly expressed and evaluated for their ability to degrade PS. Lipoprotein Lipase (LPL), Lysosomal Acid Lipase (LIPA), and Palmitoyl-Protein Thioesterase 1 (PPT1) demonstrated significant activity towards PS. Graf and co-workers claimed that whereas 1D LC-MS/MS methods detect HCPs reliably down to approximately 50 ppm, the expected threshold of the identified PS-degrading HCPs is below 10 ppb (Graf et al., 2021b). The enrichment factor of 6’000 was calculated based on the ratio of hydrolytic activity in the drug substance and the anti-CHOP elution fraction as well as on the respectively employed protein concentrations used. Hence, we suggest that using anti-HCP affinity chromatography could be a potential method for detecting and identifying trace amounts of HCPs, which may impact polysorbate degradation.

### Native digest

Huang et al. (2017) introduced a native digestion method for improved HCP identification. In this method, trypsin was added directly to the sample under non-denaturating conditions. This left the therapeutic protein intact while the low abundant HCPs were digested. As a result, the amount of antibody peptides digested is reduced compared to ones deriving from HCPs (Huang et al., 2017). Native digestion has become a key method for sample preparation in HCP characterisation using mass spectrometry due to its simplicity and speed (Kufer et al., 2019; Chen et al., 2020a; Zhang et al., 2022b). Shorter incubation times and lower trypsin-to-substrate mass ratios have been found to be beneficial when striving for an optimum digestion of all present HCPs, while maintaining the amount of digested therapeutic protein as little as possible. This resulted in an increase of peptide identifications by 67% and HCP identification by 84% (Nie et al., 2021). By fractionating the digested peptides into multiple sub-samples, the reduced complexity of the samples can further improve the sensitivity by 39%–54% for HCP identification, depending on the type of column used. However, this comes at the expense of requiring a larger sample size to be measured, which reduces sample throughput (Kufer et al., 2019). Yang et al. (2021) further modified the method by increasing the amount of substrate-to enzyme ratio, adding SDC (sodium deoxycholate) during the reduction step in order to minimize interaction of HCPs with the drug product, and adding a solid phase extraction step for optimal antibody removal. These modifications increased HCP identification rates by 10–100-fold compared to previous publications and achieved a robust sensitivity as low as 0.1 ppm (Yang et al., 2021). The combination of the HCP enrichment and separation techniques previously described, along with native digestion, can further increase the sensitivity of HCP detection (Chen et al., 2020c; Mörtstedt et al., 2020; Wang et al., 2020; Zhang et al., 2022c; Zhao et al., 2022).

### Mass spectrometry

In addition to novel methodologies for sample preparation, recent advances in applying various mass spectrometry acquisition methods have further improved the ability to detect low abundant HCPs. The most common proteomics methods include data-dependent acquisition (DDA), data-independent acquisition (DIA), and targeted MS. DDA is preferred for protein discovery and HCP identification, as it requires no prior knowledge about proteins in the sample. Peptide fragmentation is based on their abundance in MS1 survey scans. The resulting MS2 spectra from the most abundant peptides in MS1 are used for sequence database searching to create peptide spectrum matches. In DDA, quantification is based on the MS1 signals of identified peptides where intensities from different samples are compared in a relative manner (Aebersold and Mann, 2016).

DIA provides an untargeted approach for identifying HCPs by acquiring full MS2 fragment spectra over a specified mass range. Using prior knowledge in the form of a spectral library generated from previous data-dependent acquisition (DDA) runs, can be used to deconvolute the complex chimeric MS2 spectra and assign them to the best matching peptide fragmentation patterns. Typically, quantification is based on MS2 signals (Ludwig et al., 2018). New software algorithms can now generate spectral libraries directly from DIA measurements or even *in silico* from a protein database (Demichev et al., 2020; Zhang et al., 2020a). Using a library free approach or *in silico* databases, identification of very low abundant HCPs can be achieved that are normally not annotated in a spectral library based on DDA measurements. Although DIA measurements result in complex datasets that require extensive raw data analysis and interpretation, there have been instances where DIA workflows were used for quantification of specific HCPs and later validated by PRM measurements (Kreimer et al., 2017). Additionally, its usage for quantification strategies using commercially available internal standards has been shown to be applicable (Hessmann et al., 2023).

Targeted MS methods, such as selected/multiple reaction monitoring (SRM/MRM) and parallel reaction monitoring (PRM), can be employed to achieve absolute quantification of specific, selected HCPs using pre-defined transition pairs. While SRM/MRM measurements are carried out on triple quadrupole instruments, where each transition of interest needs to be acquired singly, PRM measurements using orbitrap instruments can acquire all transitions of a targeted peptide simultaneously (Peterson et al., 2012; Picotti and Aebersold, 2012). Targeted methods can greatly improve the sensitivity of HCP detection at low ppm ranges due to their high specificity, although method development requires initial time and cost investment. Additionally, the ability to retrieve absolute quantitative information from targeted methods depends on the use of internal standards, such as synthetic stable isotope labelled peptides or proteins. These standards have identical physicochemical properties compared to the corresponding endogenous peptides, ensuring precise and accurate concentrations of the HCPs of interest in a given sample. These standards need to be tested thoroughly in terms of LODs and LOQs to determine precise and accurate concentrations of the HCPs of interest. Publications over the years have shown the versatility of this method in quantifying various HCPs of interest with high sensitivity and precision. For instance, Gao et al. (2020) and Chen et al. (2021) have shown that targeted methods can be readily implemented for early and late-stage process development within a short timeframe for high-risk HCPs such as LPL, LIPA, LPLA2 and PLBL2, while achieving a high level of quantitative accuracy at concentrations ranging from 1 to 100 ppm. E. et al. (2023) were even able to develop a method that has LLOQ levels below 1 ppm for CES when combining PRM with a native digestion protocol.

Over the years, ion mobility coupled mass spectrometers have gained high interest in the field of proteomics. This technique greatly increases peptide identification rates by pre-fractionating ion packets based on their mobility in a nitrogen gas stream before entering the mass spectrometer (Swearingen and Moritz, 2012; Meier et al., 2015). For HCP analytics, this type of pre-fractionation can significantly reduce the high dynamic range between the highly abundant peptides from the drug product and trace amounts from HCPs by separating them based on ion mobility. Publications that combine High-Field Asymmetric Waveform Ion Mobility Spectrometry with native digestion, protein depletion, and data-independent acquisition methods have demonstrated the technique’s flexibility and efficacy in identifying HCPs under challenging conditions (Johnson et al., 2020; Beaumal et al., 2023).

## Activity based assays related to PS degradation

**FIGURE 4:**
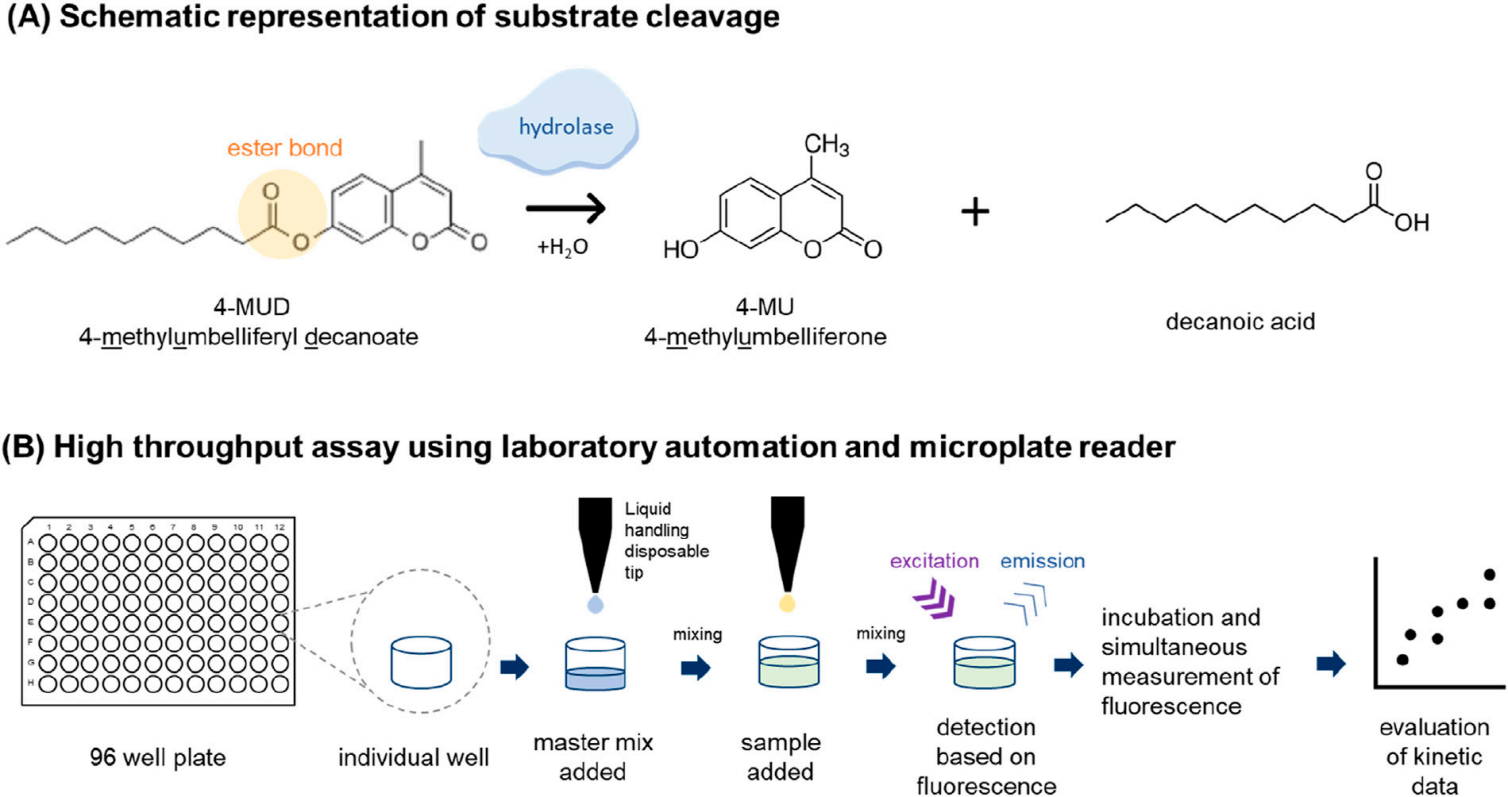
Fluorescence-based activity measurement of hydrolases using MUD4 substrate as an example. **(A)** Substrates imitating the basic structural properties of polysorbate like 4-MUD can be used to access the hydrolase activity. 4-MUD is split by the hydrolase (not scaled) at the ester bond resulting in 4-methylumbelliferone (4-MU) and decanoic acid as a product. The 4-MU can be detected based on the fluorescence. **(B)** The most straightforward way to perform the assay is to use laboratory automation and microtiter plates like a 96 well plate. The master mix is added by a multichannel arm containing 4-MUD along with assay buffer. Afterward, the samples are added, and the content of each well is mixed. The hydrolases present in the samples will start to cleave the 4-MUD and as a result 4-MU is produced. After placing the microtiter plate into a microplate reader, the wells are read at regular intervals. As a result, a kinetic curve is obtained where the fluorescence of the 4-MU is displayed as a function of time. The steeper the curve, the more hydrolase activity is present in the sample. *For references see text.*

Lipolytic assays that stimulate the degradation of PS are useful for monitoring lipolytic activity during upstream and downstream process development, especially if these assays can be conducted in a high-throughput format (Figure 4). Accordingly, several assays have been published quantifying lipolytic activity by enzymatic release of a fluorescent product, 4-Methylumbelliferone, which is esterified within the initial non-fluorogenic substrates (Jahn et al., 2020). The major difference of the published assays lies in the carboxylic acid moiety coupled to 4-Methylumbelliferyl, e.g., caprylic acid (C8, Bhargava et al., 2021) or oleic acid [unsaturated C18, (Jahn et al., 2020)]. It has to be kept in mind that each of these substrates only mimic a part of the heterogeneity of either PS20 or PS80. In addition to these fluorogenic assays, an assay based on electrochemiluminescence has also been published (Gupta et al., 2023). Though theses assays are crucial for process optimization, they cannot identify the specific HCPs which are responsible for PS degradation without prior heterologous expression and purification of protein candidates of interest. However, this approach is rather labour-intensive, time-consuming, and carries the risk of active enzyme contamination (Zhang et al., 2020b).

A smart alternative to this approach is a technique called Activity-Based Protein Profiling (ABPP), a technique that combines the power of mass spectrometry with enzymatic activity. ABPP was invented by Cravatt and co-workers and was initially used for functional proteomics studies (Adam et al., 2002). The centrepiece of ABPP is a bi-specific probe containing a molecule part which irreversibly binds to serine hydrolases as well as a molecule part that can be used for affinity enrichment, such as a biotin or desthiobiotin group. The two molecule parts are connected by a flexible linker. Enzymes bound to the probe can be analysed by LC-MS after affinity enrichment, assuming specificity of the ABPP probe towards the enzyme class (es) of interest. Consequently, ABPP on the one hand is an enrichment technique, on the other hand it´s highest potential lies in obtained information on enzymatic activity.

Zhang et al. (2020c) were the first to use activity-based protein profiling (ABPP) to tackle enzymes responsible for polysorbate degradation. They used a commercially available FP-desthiobiotin probe and identified the Liver carboxylesterase B-1-like protein (CES-B1L) and the Liver carboxylesterase 1-like protein (CES-1L) as the primary contributors to PS80 degradation in a mAb drug produt. Li et al. (2021) used two chemical probes, FP-biotin and FP-Desthiobiotin, to enrich and characterise HCPs that are active towards polysorbates (Li et al., 2021). The authors found that the FP-biotin probe was more effective for most serine hydrolases compared to the FP-desthiobiotin probe. Additionally, they discovered a new lipase, phospholipase A2 group VII (PLA2G7/LPLA2), which can induce PS80 degradation. Liu et al. (2022) optimised the technology by developing a new ABPP probe, which was designed to mimic the structure of polysorbate and thus to more specifically target lipases involved in polysorbate degradation. They found that the newly synthesized probe was more effective in enriching lipases and serine hydrolases than commercially available FP-biotin and FP-desthiobiotin. In analogy to Liu et al. (2022), a novel ABPP probe was developed by Dehghani et al. (2023). The probe is based on the anti-obesity drug orlistat, which has been shown to be an efficient inhibitor for enzymatic polysorbate degradation in this and other publications (Jahn et al., 2020; Graf et al., 2021b; Dehghani et al., 2023). According to the studies of Li et al. (2021) and Liu et al. (2022) also Dehghani et al. (2023) showed that specificity of the applied ABPP probes is key to really tackle the enzymes of interest and that it is beneficial to have more the one ABPP probe in hand to investigate the enzymes involved in PS degradation. Furthermore, Dehghani and colleagues (2023) demonstrated that the conditions under which an ABPP experiment is conducted have to be carefully selected to reflect the conditions of the biopharmaceutical matrix of interest, e.g., the final drug product. Previously published ABPP experiments were conducted at neutral or slightly alkaline pH (Zhang et al., 2020c; Li et al., 2021; Liu et al., 2022). However, Dehghani et al. (2023) conducted the ABPP experiments using an Orlistat-based probe and FP-desthiobiotin both at acidic (pH 5.5) and alkaline (pH 8) pH levels, respectively. They observed different enzyme enrichment behaviour under both conditions. It should be noted that this also provides an opportunity to characterise PS degradation through ABPP experiments. This method allows for the screening of active enzymes under various conditions, such as the addition of selected trace metal ions.

Recently, Šprager et al. (2024), using an ABPP assay were able to identify acyl-protein thioesterase-1 in a monoclonal antibody formulation that degrades polysorbate 20/80 (multi-compendial grade). In the presented case study, the authors discuss the thioesterase-1 being co-purified with the specific antibody via a hitchhiking mechanism (Šprager et al., 2024).

Finally, as ABPP probes are designed to target serine hydrolases, it could be argued that the technique may not detect enzymes that act via a different catalytic site, such as those that utilise histidine (Li et al., 2022). Therefore, it is important to check in a pre-experiment whether the applied probe can completely inhibit PS degradation. It is important to keep in mind that enzymes identified as active through an ABPP experiment may not necessarily be acting on polysorbates, depending on the specificity of the ABPP probe (Li et al., 2022).

An overview of activity-based assays that can be used to determine the propensity of polysorbate degradation of a sample or individual HCPs is shown in Figure 4.

## Polysorbate degrading enzymes: current, scientific knowledge from literature

This chapter summarises the current scientific knowledge on the most relevant enzymes that degrade PS. In recent years, our understanding has increased significantly due to: (i) improvements in detecting HCPs in particular activity-based mass spectrometry profiling, (ii) expression studies of recombinant enzymes, (iii) increased use of specific inhibitors, and (iv) characterisation of PS degradation profiles.

PLBL2 was initially described as a PS degrading enzyme in 2015, when recombinant human PLBL2 was spiked in a drug product and the PS degradation was found to be accelerated (Dixit et al., 2016). However, more recent studies utilizing multiple approaches have provided compelling evidence contradicting the involvement of PLBL2 in polysorbate degradation (Zhang et al., 2020b). This is a nice example of how scientific data should be evaluated independently making sure that correct conclusions are made. The list of enzymes listed in Table 5 is not complete, rather aims to focus on critical and well characterized HCPs as examples to present the complexity of assessing the activity and effect of these HCPs.

Given the potential consequences of PS degradation on patient safety, it is crucial to publish scientific work on HCPs conducted within biopharmaceutical organisations. Sharing results and methodologies will contribute to the collective knowledge in this field and promote informed decision-making in drug development projects. It is of importance to share the results utilizing scientific standards–for example, releasing a list of HCPs without a unique identifier for the protein like Uniprot accession number might lead to misunderstandings. The scientific data on PS degrading HCPs is increasing exponentially (Wuchner et al., 2022b)–articles summarizing the current scientific knowledge (Jones et al., 2021) will be ever more important. Furthermore, databases that are aiming to summarize the relevant information regarding critical HCPs related to biologics will be pivotal in the future, like the “Host Cell Proteins Data Platform” of BioPhorum.

### Carboxylesterases (CES1 | CES1F | CES1-B1L | CES2C)

Carboxylesterases (CES) belong to the serine hydrolase superfamily and are located intracellularly within the lumen of the endoplasmic reticulum lumen (Sanghani et al., 2009). CES enzymes catalyse the ester cleavage of a large number of structurally diverse ester- or amide-containing substrates into the corresponding alcohol and carboxylic acid (Wang et al., 2018). Various CES proteins are present in humans, each with a specific tissue residence [e.g., CES-1 is predominantly found in the liver and CES-2 in the small intestine (Sterri and Fonnum, 2009) (Chapter 68), (IMAI, 2006)], and substrate preferences. For instance, CES-1 enzymes preferentially hydrolyse esters with a small alcohol and a large acyl group while CES-2 enzymes prefer esters with a large alcohol group (Sterri and Fonnum, 2009). There is strong evidence that carboxylesterases play a critical role in the observed PS degradation phenomenon. Different CES enzymes derived from CHO cells, including CES-1, CES-1F, CES-B1L and CES-2c, have been detected in mAb drug products that exhibit PS instability. Experimental confirmation of their activity towards PS degradation has been reported (Zhang et al., 2020c; Zhang et al., 2022c; Gupta et al., 2023; Kovner et al., 2023; Maier et al., 2024b). This makes the mentioned CES proteins particularly interesting targets for process optimisation efforts as well as for gene knockout strategies in CHO host cells. In CHO cells, the molecular masses of these four CES proteins are 98 kDa (CES1), 65 kDa (CES-1F), 78 kDa (CES-B1L) and 65 kDa (CES-2c).

### Isoamyl acetate-hydrolysing esterase 1 (IAH1)

The isoamyl acetate-hydrolysing esterase 1, which is encoded by the Iah1 gene, represents an enzyme that catalyses the hydrolytic cleavage of acetyl-esters (Ma et al., 2015). In CHO cells, the protein has a molecular mass of 31 kDa and belongs to the GDSL (Gly-Asp-Ser-Leu) lipolytic enzyme family due to a specific GDSL motif present near the N-terminus. GDSL esterases and lipases are hydrolytic enzymes exhibiting multifunctional properties such as broad substrate specificity and regiospecificity (Akoh et al., 2004). IAH1 has not yet been extensively researched in human and CHO cells, so its functional cellular role of this enzyme in these cell types is not well understood. However, Kovner and co-workers (Kovner et al., 2023) and Maier and colleagues (Maier et al., 2024b) characterised the recombinantly expressed IAH1 enzyme for its ability to degrade PS20 and PS80 formulations after it was identified as a HCP contaminant in drug product formulations. Both groups concluded that although at high concentration IAH1 was shown to be able to degrade PS20, the degradation kinetics were found to be much slower compared to other recombinantly expressed lipases/hydrolases (Kovner et al., 2023), and that fact that the protein mainly resides in cells reduces the risk of appearance in DPs. In CHO cells, IAH1 seems to be mainly present intracellularly (Maier et al., 2024b). This feature in conjunction with its comparatively slow PS20 degradation activity reduces the risk profile of this hydrolase towards PS degradation in biopharmaceutical drug products, as compared to other suspect lipases.

### Lipase A (LIPA | lysosomal acid lipase LAL)

Lipase A (LIPA) is an essential enzyme in humans. It hydrolyses cholesteryl ester and triglyceride delivered to the lysosome under acidic conditions (Sando and Rosenbaum, 1985; Zhang, 2018), therefore it is an intracellular protein. The first report indicating LIPA in the context of PS degradation in a biological product stems from 2021 (Graf et al., 2021b). In this study, HCPs were enriched by utilizing both protein-A and anti-HCP affinity chromatography and analysed the generated samples by mass spectrometry. A number of enzymes were recombinantly expressed and purified, and subsequently were incubated with PS20 or PS80. LIPA exhibited activity against both PS types at a concentration of 10 μg/mL, indicating its efficacy as a PS degrading enzyme. In the study by Zhang et al. (2022b), it was clearly indicated that in a product containing both LPL and LIPA, the latter was responsible for the degradation. The use of recombinant enzymes combined, with specific inhibitors allowed to draw a clear scientific conclusion: LIPA has a strong hydrolytic activity at low concentrations and showed a unique degradation pattern. However, due to its tendency to release more insoluble fatty acids from PS20, this enzyme is associated with a higher risk of PS degradation. These conclusions were confirmed in systematic recombinant lipase expression studies published by Kovner et al. (2023) and Maier et al. (2024b).

### Lipoprotein lipase (LPL)

Lipoprotein Lipase (LPL, EC 3.1.1.34) is one of the best characterized lipases related to PS degradation. It was first identified over 60 years ago by Korn (1955a) and Korn (1955b). LPL belongs to the pancreatic lipase gene family (Carrière et al., 1998), whose members have triglyceride lipase activity (EC 3.1.1.3) and are all closely related to LPL, as indicated by strong sequence conservation (MacDonald et al., 2018). Five of the proteins express the conserved and well-described catalytic triad of LPL (Emmerich et al., 1992) and are also expressed in CHO cells. LPL was identified as an HCP impurity already a decade ago in two-dimensional liquid chromatography coupled with high-resolution mass spectrometry (Doneanu et al., 2012). Levy et al. (2016) identified LPL as a difficult-to-remove protein among CHO HCP impurities, representing a small sub-population within a larger group. Chiu et al. (2017) demonstrated that recombinant LPL exhibits enzymatic activity against PS80 and PS20 under solution conditions commonly found in mAb formulations. The cell culture harvest fluid from LPL knockout CHO cells showed a significant reduction in polysorbate degradation compared to wild-type samples, without negatively impacting cell viability. In a recent publication Zhang et al. (2022b) identified LAL and LPL in various drug products. To clarify which lipase is responsible for the PS degradation, they utilised recombinant proteins. Through the use of specific inhibitors, they identified LAL as the root cause. In several drug products containing similar levels of LPL (0.5–1.5 ppm), no PS degradation was observed when LAL was inhibited. This indicates that at levels under 1.5 ppm, LPL may not be considered critical for PS degradation.

The CHO LPL protein shares 93.47% identity with its human ortholog (MacDonald et al., 2018). This high level of conservation allows for the transfer of scientific data from the human protein to the (Levy et al., 2016) CHO ortholog. In certain studies, recombinant human LPL (Graf et al., 2021b; Kovner et al., 2023) or other species such as *Burkholderia* sp. (Glücklich et al., 2021) have also been used. Despite intensive research, the structure of LPL remained elusive for a long time. However, in 2019, Birrane et al. (2018) and Arora et al. (2019) resolved it after discovering that GPIHBP stabilises the LPL protein structure (Mysling et al., 2016). The crystal structure revealed that LPL is active as a monomer when complexed with GPIHBP1, as supported by density gradient ultracentrifugation (Beigneux et al., 2019). The hydrolase domain of LPL has a tendency to spontaneously unfold, leading to protein destabilisation. The multiple heparin-binding domains of LPL merge into a continuous basic patch. Binding of this domain with the acidic domain of GPIHBP1 leads to molecule stabilisation. The crystal structure supports the previous assumption of head-to-tail homodimerization of LPL. This is further underpinned by SAXS data, which indicates that dimers also exist in solution (Birrane et al., 2018). This raises an interesting question of whether LPL stabilisation occurs either through dimerization or by interacting with other HCPs or even with the therapeutic protein. The latter could potentially explain how an unstable molecule can persist through the wide pH ranges of a downstream purification process. The interactions of LPL should be studied in more detail, particularly focusing on possible hitchhiking scenarios, as described in the recent work of Hecht et al. (2022). This work highlights low-affinity interactions of PLBL2 and LPLA2 with the CH1 domain of IgG1 and IgG4 molecules.

### Lysophospholipase-like protein 1 and 2 (LYPLAL1 and LYPLAL 2)

LYPLAL 1 and 2 were identified in the ABPP paper of Li et al. (2021) and LYPLAL 1 was also recombinantly expressed in the systematic recombinant lipase expression study by Kovner et al. (2023). Enzymatic activity was measured against both fluorometric substrate and PS20, but only at extremely high concentrations of the recombinant enzyme. According to Bürger et al. (2012), the protein in humans has hydrolytic activity towards short chain substrates due to its shallow active site. It does not exhibit phospholipase or triacylglycerol lipase activity. Therefore, it is unlikely that LYPLAL1 and LYPLAL2 play a significant role in PS degradation. The intracellular localization of the human ortholog is cytoplasmic (Tian et al., 2012).

### Palmitoyl-protein thioesterase 1 and 2 (PPT1 and PPT2)

PPT1 was discovered in a highly enriched purified sample of a monoclonal antibody (mAb). Subsequent recombinant protein expression confirmed its activity against PS (Graf et al., 2021b). PPT1 is a thioester hydrolase (EC 3.1.2) that acts on ester bonds and is related to carboxylic ester hydrolases that are also indicated in PS degradation such as LPLA2, LPL or CES-1L. However, the fact that a thioester hydrolase is also able to degrade PS, suggests that other thioesters might be also causing PS stability issues in drug substance. The PPT1 protein localizes intracellularly to the nucleus, Golgi- and endoplasmic reticulum but also to the extracellular space (Uniprot G3HN89). PPT2 was identified in the ABPP study conducted by Li et al. (2022) suggesting its potential ability to degrade PS. Hecht et al. (2022) reported ultra-low affinity interaction data for PPT1 and mAbs, but this interaction was only observed in a native MS experiment. Maier and co-workers (2024b) further confirmed the PS degrading activity of PPT1 by recombinant expression of the CHO PPT1 protein and subsequent characterisation (Maier et al., 2024b). The weak and concentration-dependent binding effect may be mediated by electrostatic repulsion. In a subsequent publication, the PS degrading activity of recombinant PPT1 was confirmed. Furthermore, an acidic pH optimum of pH 4-5 was identified (Gupta et al., 2023).

### Phospholipase A1 member A (PLA1A)

PLA1A is a homolog of LPL and the enzyme is endogenously expressed in CHO cells (MacDonald et al., 2018). PLA1A belongs to the pancreatic lipase gene family (EC 3.1.1.3) and it has a triglyceride lipase activity similar to other members of this enzyme family comprising, e.g., LPL, LIPG, LIPC or PNLIP. The localization of the protein is extracellular. These lipases also share strong conservation of amino acids around their active centre, especially for the serine- and aspartic acid moieties, while the conservation is less pronounced around the histidine residue of the catalytic tirade. In the systematic expression paper by Kovner and co-workers (Kovner et al., 2023), this lipase was also expressed recombinantly, and its activity towards PS degradation was confirmed by both fluorometric esterase activity and PS20 assay. However, a relatively large amount of the enzyme was necessary to detect a notable PS degradation.

### Platelet-activating factor acetylhydrolase (PLA2G7 | PAF-AH)

Platelet-activating factor acetylhydrolase (PAF-AH/PLA2G7) is a human enzyme that belongs to the phospholipase A2 (PLA2) hydrolase group. It activates the platelet-activating factor, a pro-inflammatory phospholipid produced by activated platelets (Tjoelker et al., 1995). PAF-AH/PLA2G7 is involved in phospholipid catabolism during inflammation and oxidative stress response. It specifically targets phospholipids with a short-chain fatty acyl group at the sn-2 position (Stremler et al., 1991). Phospholipases are one of the earliest identified and studied enzyme activities. The PLA2 superfamily can be traced back to the discovery of lytic actions of snake venom at the end of the 19th century (Dennis et al., 2011). The PLA2 superfamily has been classified into 16 groups (groups I to XVI) based on the chronology of their discovery (Khan and Ilies, 2023). The members of these PLA2 groups can be classified into six subfamilies based on their location in the body, substrate specificity and differences in their physiological function (Khan and Ilies, 2023). PLA2 enzymes can exist in various forms, including secreted, cytosolic, calcium-independent and lipid lipoprotein-associated forms (Shayman et al., 2011). PAF-AH PLA2s thereby belong to the groups VII and VIII of the PLA2 superfamily (Dennis et al., 2011). PAF-AH is a 50 kDa serine-dependent hydrolase that possesses a lipase motif and is extensively glycosylated (Tew et al., 1996). At least two different forms of PAF-AH are known, an intracellular as well as a secreted form that circulates in the plasma of humans (Tjoelker et al., 1995). As with other organisms, PAF-AH is a secreted protein also in CHO cells (Maier et al., 2024b) and therefore it is present in the harvested cell culture fluid (HCCF) at the end of the therapeutic protein production process. Although PAF-AH and its gene function have not been specifically investigated in CHO cells, a recent study by Li and colleagues (Li et al., 2021) identified PAF-AH as a HCP contaminant in CHO cell-derived biopharmaceutical drug products using an activity-based protein profiling (ABPP) approach. The study further confirmed the high activity of the enzyme in degrading polysorbates (Li et al., 2021), which was recently confirmed by Maier et al. (2024b). The combination of this feature with the active cellular secretion, as well as the potential co-elution with monoclonal antibodies during the downstream purification process, makes PAF-AH a very critical HCP for PS degradation and particle formation.

### Lysosomal phospholipase A2 (PLA2G15 | LPLA2)

Lysosomal phospholipase A2 (LPLA2 or PLA2G15) belongs to the same phospholipase A2 superfamily, which also includes PLA2G7 (PAF-AH). LPLA2 proteins are the members of group XV of the PLA2 superfamily, known to be localized on intracellular vesicles such as lysosomes and endosomes (Ma and Turk, 2001). The protein has a molecular weight of 47 kDa and, although it was previously thought to be located only intracellularly (lysosomes and endosomes), recent studies have shown that it is at least partly secreted by CHO cells (Hall et al., 2016). The presence of LPLA2 in CHO cell derived therapeutic antibody drugs has been reported to play a key role in polysorbate degradation (Hall et al., 2016). Hall et al. (2016) reported that the presence of LPLA2 in CHO cell-derived therapeutic antibody drugs plays a crucial role in polysorbate degradation. The authors identified LPLA2 proteins at sub-ppm levels (<1 ppm) in three different CHO cell-derived mAb products that exhibited polysorbate instability upon storage of the drug product. However, in a fourth mAb formulation that did not show polysorbate degradation, the presence of LPLA2 could not be detected. Furthermore, the authors recombinantly expressed LPLA2 in CHO cells and demonstrated that the purified enzyme can hydrolyse PS20 and PS80. Hall et al. (2016) also noted that LPLA2 exhibits notable differences in the hydrolysis profiles of PS20 and PS80. They found that LPLA2 prefers the free form of polysorbates in solution while polysorbates in the micelle form should resist LPLA2 mediated degradation (Hall et al., 2016). Hall et al. (2016) showed that LPLA2 binds to the CH1 domain of antibodies through ultra-low affinity interaction. Eventually, Maier and co-workers demonstrated the PS degradation activity of the purified recombinant LPLA2 from CHO cells (Maier et al., 2024b), which underlines the criticality of this enzyme for PS degradation in DPs.

### Sialic acid acetylesterase (SIAE)

The human SIAE removes acetyl moieties from the sialic acid in position 9 and 4; the protein is glycosylated. The glycosylation status affects the biological activity of the enzyme and essential for the release of the protein into the cell culture medium in cell lines (Orizio et al., 2015). SIAE was characterized by recombinant expression, quantification in mAb formulations, and by depletion studies (Zhang et al., 2021). The recombinant enzyme showed a strong PS20 degrading activity, with a specific degradation fingerprint targeting shorter monoesters. Based on the ester distribution and free fatty acid solubility of Doshi et al., SIAE poses a lower risk of particle formation than other lipases (Doshi et al., 2015). This observation is further supported by the fact that SIAE did not exhibit significant degradation activity below 1 ppm. It is possible to evaluate whether switching to PS80 would be beneficial for affected formulations to achieve PS stability, as the enzyme SIAE degrades PS20 but not PS80 as strongly.

Notably, what makes this enzyme and the study of Zhang et al. (2021) of particular interest, is the fact that SIAE belongs to the SGNH-hydrolase family and as an esterase it removes acetyl units from sialic acid. The PS degrading activity of the enzyme is therefore surprising, although the publication suggests that the structural similarity between sialic acid and the PS POE head group is the underlying reason for the PS degradation. This beautiful example highlights the importance of carefully evaluating HCP contaminant data and pursuing recombinant expression and further characterisation, even if the enzyme classification is not directly pointing towards a PS degrading activity. The observations regarding the activity of SIAE were also supported by the results of Kovner et al. (2023), but were partially contradicted by Gupta et al. (2023). It was noted that different enzyme preparations were used in these studies. The glycosylation status of the SIAE enzyme plays an important role for its biological function, and therefore, the discrepancy can likely be attributed to these differences.

### Sphingomyelin phosphodiesterase 1 (SMPD1)

Sphingomyelin phosphodiesterase, encoded by the Smpd1 gene, represents an enzyme that hydrolyses the membrane lipid sphingomyelin to phosphorylcholine and the bioactive lipid ceramide (Smith and Schuchman, 2008). This protein and its function have also not been specifically investigated in CHO cells yet. In humans, the Smpd1 gene gives rise to two different protein forms (acid sphingomyelinase, ASM). One form, ASM-L, is found in the lysosome, while the other, ASM-S, is secreted (Schissel et al., 1996; Kornhuber et al., 2015). Mutations in the SMPD1 gene cause Niemann-Pick disease (NPD), which has two forms (type A and B) and is a lysosomal storage disorder (Smith and Schuchman, 2008). In CHO cells, only the 66 kDa secreted form of SMPD1 has been reported. This protein has been recently identified as another difficult-to-remove HCP, as it has been shown to contaminate biopharmaceutical drug products (Kovner et al., 2023). Unlike IAH1 and LIPA, which are mainly found intracellularly, recent experimental data suggests that SMPD1 is actively secreted into the supernatant by CHO cells and therefore contaminates the HCCF, from which the therapeutic protein is later being purified (Maier et al., 2024b). It has been reported that exposure of cells or animals to stress frequently induces ASM-mediated ceramide production that leads to cell death (Smith and Schuchman, 2008). In this context, cell stress is often observed in CHO cells during the upstream production process, especially towards the end of a fed-batch cultivation due to the accumulation of cytotoxic metabolites and nutrient depletion. This could account for the presence of SMPD1 in CHO cell-derived HCCF and, consequently, in the resulting drug product after purification and formulation. Despite being actively secreted and potentially forced to secrete under stressed culture conditions, SMPD1 has been found to have no PS20 degradation activity (Maier et al., 2024b). Therefore, SMPD1 is not considered to be a major driver of PS degradation and particle formation in CHO cell derived biopharmaceutical drug products.

## Mitigation strategies for polysorbate degradation

### Removal of PS degrading enzymes by cell line engineering

Cell line engineering has become a powerful tool to rationally tailoring biopharmaceutical production cell lines (Fischer et al., 2015; Jazayeri et al., 2018). Regarding the removal of critical HCPs either directly in a host or production cell line or during the bioprocess (e.g., downstream purification), there is plenty of literature available (Shukla and Hinckley, 2008; Yumioka et al., 2010; Brodsky et al., 2012; Hogwood et al., 2014; Chollangi et al., 2015; Chiu et al., 2017; Laux et al., 2018; Fukuda et al., 2019; Li et al., 2019; Kol et al., 2020; Dovgan et al., 2021). However, information on the specific removal of lipases and hydrolases is limited (Chiu et al., 2017). For instance, Chiu and co-workers demonstrated that genomic knock-out (KO) of a specific lipase (LPL) can eliminate the cell line’s PS degrading properties (Chiu et al., 2017). There are few examples in the literature of genomic knockouts of PS-degrading enzymes, likely due to the potential for identifying a PS-degrading HCP. It is important to experimentally determine which enzymes can be knocked out and which are essential for the cell to function as an efficient cell factory, given the potential native functionalities of lipases and hydrolases in a cell. In addition, as discussed in the above section, it is fundamental to have a reliable list of target lipases at hand for specific host cell line engineering strategies. The availability of such a list including confirmed active PS degrading lipases/hydrolases is crucial before endeavours to perform (multiple) genomic knockouts and the accompanying tedious cellular and bioprocess characterizations will be pursued. Before conducting genetic engineering approaches, it is very important to have a thorough understanding of the genomic sequence of the host cell line to be engineered. This ensures that the process is conducted accurately and efficiently. The CHO-K1 cell line is the most used mammalian expression system for industrial production of therapeutic glycoproteins. In 2011, Xu et al. (2011) published the first whole genome sequence assembly of this cell line, followed by the genomic sequence of the Chinese hamster in 2013 by Brinkrolf et al. (2013).

Since then, many industrial organizations have sequenced their in-house CHO cell lines. This is because this cell type has been shown to exhibit a particularly plastic genome, which leads to a pronounced genetic heterogeneity among the different CHO host cell lines used in various laboratories (Wurm and Wurm, 2021). Whole genome sequencing data combined with refined bioinformatics analyses, are critical for identifying the genomic locations of the target genes. This information is necessary to rationally design precise genome editing tools for gene knockout strategies (MacDonald et al., 2018), such as zinc-finger nucleases (ZFNs), TALENs or RNA-guided nucleases like CRISPR/Cas9 or comparable technologies. A precisely sequenced host cell genome is also important for careful off-target screening and analysis to select the most suitable and efficient genome editing tools (Lee et al., 2015). The tool can also be used for an *in silico* prediction of potential lipases or hydrolases that have not yet been characterized, for example, through Pfam protein motif classification (Sonnhammer et al., 1997). The use of a precisely sequenced and annotated host cell genome can improve the accuracy of mass spectrometry-based HCP identification in drug products. This is because the predicted protein sequences of the entire proteome are derived from the genomic nucleotide sequence of exactly that host cell line (Baik and Lee, 2014; Lee et al., 2015).

**FIGURE 5:**
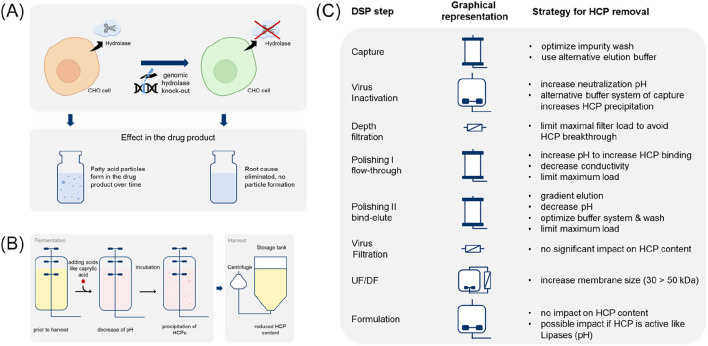
Mitigation strategies for the reduction of PS-degrading HCPs in DPs. **(A)** Cell line development offers a potent solution for complete elimination of PS-degrading HCPs by utilizing genomic knock-outs. Critical hydrolases that often persist by the end of the purification process can be specifically eliminated. The genomic knock-out disrupts the coding sequence of the desired hydrolase rendering the resuting protein functionally inactive. Hence, if specific hydrolases were the root cause of particle formation in a given bioprocess, no particle formation will be expected after the genomic knock-out of these hydrolases. **(B)** At the end of the cell culture fermentation process prior to harvest, the pH can be downshifted followed by a short incubation period. This process step will lead to a precipitation of impurities that can be eliminated during the cell culture harvest procedure eventually leading to a lower HCP level in the harvested cell culture fluid (HCCF) that is being handed over to the Downstream processing unit operations. However, this acidic harvesting procedure must be optimized as it can also lead to lower process yields. **(C)** Downstream processing and formulation offers a series of impurity removal strategies, they are sorted according to the sequence of the bioprocess and summarized as a short list. For references see text.

Genomic knockout of critical PS degrading lipases/hydrolases that degrade PS can provide a long-term solution to the enzyme-mediated hydrolytic degradation of polysorbates in biopharmaceutical drug products (Figure 5A). Removing the critical HCPs directly tackles the root cause. However, despite substantial technological advancements in host cell engineering, such as the discovery and refinement of precise genome editing tools, experimental approaches remain complex and require time-consuming and laborious efforts to establish and characterize host cells after the respective gene knockout(s). These efforts involve comprehensive genetic and phenotypic characterisation of the engineered host cell lines, as well as investigations into the product quality of the recombinantly expressed therapeutic proteins. The aim is to demonstrate that the modified cell lines are suitable for the clinical and commercial manufacturing of biotherapeutics. In this context, a recent study by (Weiß et al., 2024) presented a new method for multi-lipase gene knockdown using artificially designed microRNAs (amiRNAs). The method induced simultaneous reduction of several critical PS degrading enzymes in the production cell line (Weiß et al., 2024). Certainly, such a novel approach would omit generating stable knockout host cell line and the subsequent tedious host cell line characterisation process.

Though the establishment of genetically engineered CHO host cell lines with multiple hydrolases silenced is a complex and tedious endeavour, it might represent the most promising long-term solution for a PS degradation free expression platform that applicable to a wide range of different molecule formats.

### Removal of PS degrading enzymes by bioprocess engineering

With regards to removal during the upstream or downstream purification process, scientific work is actively underway, but not much data has been published to date. This ongoing work may reveal whether the PS-degrading enzymes behave differently from other HCPs for which extensive knowledge is already available. It is important to note that the information currently available is mainly focusing on the mammalian expression systems, and more specifically on CHO cells, where HCP monitoring is an important cornerstone to ensure patient safety, and CHO cells represent by far the most widely used expression system for the production of therapeutic glycoproteins. In particular, there is less knowledge on enzyme-mediated PS degradation in microbial expression systems, presumably because PS degradation issues similar to those associated with CHO cells have not yet been described. However, this does not necessarily mean that PS degradation will not be an issue in the future for products produced using microbial expression systems.

There are two basic mechanisms by which HCPs can persist through the bioprocess and end up in the final drug product (Jones et al., 2021): (i) either by co-purification with the therapeutic protein or (ii) by hitchhiking on the therapeutic protein. In the case of co-purification, the physicochemical properties of the HCP are similar to the protein of interest, and therefore it co-purifies during the bioprocess (Levy et al., 2014; Levy et al., 2016). In the case of hitchhiking, the HCP binds non-specifically to the therapeutic protein and therefore is “pulled” through the bioprocess (Aboulaich et al., 2014; Levy et al., 2014; Valente et al., 2018). This is based on weak interactions that are specific for the therapeutic protein and the HCP (Hecht et al., 2022). As a result, product-specific purification development is often required, especially for complex antibody formats where the increased surface area of these molecules allows for more interactions. As specific purification development is tedious and time-consuming, it has a critical impact on the development timeline of a biological drug and the amount of resources required to develop that drug. Recently, another mechanism has been proposed by Oh and colleagues (Oh et al., 2022). Evidence provided from seven mAb processes indicated, that some proteins–especially related to unfolded protein response–are present in the high molecular weight aggregates species. These HCPs are also masked from analytical detection. The provided evidence is titillating and indicates the complexity of process development–where multiple impurity removal has to be optimized simultaneously.

In general, the overall strategy is to reduce the HCP load as early and as accurately as possible throughout the bioprocess. However, a fine balance between different critical quality attributes (cQAs) must be defined: for a given DSP process step, it is often not possible to optimise yield, aggregate and HCP removal simultaneously and compromises must be made.

As already discussed in the previous section, during the cell line development (CLD), process, critical (but not essential) HCPs for the expression system itself can be knocked out using precise genome editing technologies (Figure 5A). This results in the long-term removal of these critical lipases/hydrolase(s) and thus eliminates PS degradation mediated by these particular enzymes. For specific lipases/hydrolases that cannot be silenced or knocked out (e.g., due to their essentiality in the cell or because companies may risk violating existing intellectual property rights), tailor-made cell line selection strategies can be developed. Here, the transcriptomic heterogeneity between different clonal production cell line candidates can be exploited, leading to variations in the expression profile of specific genes and thus HCPs, and thereby might enable the selection of superior cell lines with favourable HCP profiles. However, this requires the availability of highly accurate and sensitive MS-based quantification methods for the rapid detection of specific HCP species in HCCF to enable the deselection of production cell line candidates with high expression of these HCPs. Such HCP monitoring will also need to be completed before final clone decisions are made in a development project, in order to take the results into account. Finally, if such highly specific HCP monitoring systems are not available, it is also possible to use lipase/hydrolase activity assays to screen different clonal production cell line candidates for their enzymatic activity. However, although this may reduce the risk of PS degradation potential of the production cell line, it remains a rather non-specific analysis with relatively low sensitivity. Furthermore, enzymatic activity assays that provide rapid results, as required for rapid decision-making during cell line development, may be less sensitive and therefore less useful than more elaborate assays that require more time to provide interpreted results.

The impact of different cell lines or the specific upstream process on the HCP populations is not fully understood. Recent data indicates that when using a company cell line, either null or transfected with an expression vector, the proportion of HCPs is approximately 80% with a 15% reduction when other external cell lines are used (Wright et al., 2023). The differences in the HCP profile that occur during clone selection need to be investigated in more detail, but Hamaker et al. (2022) recently showed that HCP profiles can change over time during cultivation of a CHO cell line. Of the 1'500 HCPs identified, 13% showed variable expression over time, suggesting that the HCP profile is dynamic during cell ageing and may therefore also affect the abundance of critical HCPs such as PS-degrading enzymes in the harvested cell culture fluid (HCCF) as a function of cell age.

In addition, a significant number of lipases/hydrolases are actively secreted by the cells into the supernatant during fermentation. Consequently, although high cell viability at the end of the production process is generally desirable in a bioprocess, it may only marginally contribute to a reduced abundance of critical lipases/hydrolases in the HCCF and thus to a mitigation of enzyme-mediated PS degradation, as it would only affect enzymes that are predominantly reside intracellularly. After operation of the cell culture harvesting unit operation, the total amount of HCPs can be reduced by shifting the pH to an acidic range with, for example, caprylic acid, which leads to precipitation of a part of the HCPs (acidic harvest) (LYDERSEN et al., 1994; Brodsky et al., 2012; Gupta et al., 2020) (Figure 5B). Notably, this process adaptation can only be applied if the therapeutic molecule remains stable and does not precipitate under acidic harvest conditions.

Most downstream mammalian bioprocesses use three chromatographic steps based on different chromatographic principles (Bergemann et al., 2010). This chromatographic variability is essential to achieve strong HCP removal in the bioprocess (Figure 5C). In the capture step, therapeutic monoclonal antibodies are commonly captured by a protein A ligand that binds the Fc region of the antibody. In this bind-elute step, the most effective HCP removal option is the “impurity wash,” which is often developed in a project-specific manner (Shukla and Hinckley, 2008; Yumioka et al., 2010; Li, 2017). In the next step, the pH is shifted to the acidic range (around pH 3.5) to inactivate the viruses, followed by a neutralisation step (Jacobi et al., 2014). During this neutralization, many of the HCPs precipitate due to the pH change and the pH of this step has a strong effect on HCP removal and needs to be optimised in terms of the therapeutic protein recovery (Chollangi et al., 2015). The mechanism of this precipitation is described by Greene and co-workers (Greene et al., 2018), however more relevant work would be advantageous to understand how the precipitation in this step could be enhanced and improved. Not only the pH, but also the buffer system affects the precipitation in this DSP step. If in the capture step glycine is used as an elution buffer rather than acetate, no differences will be observed in the capture product pool in terms of product quality. However, after the acid treatment and during the neutralization step, more impurities will precipitate - partially due to the lower conductivity that the glycine buffer system provides (Lakatos et al., 2024). This is ever more important in process development–do not only optimize and focus on individual steps but think about the whole bioprocess. Some decisions will affect not only the current step but will have important effects in later unit operations. After the depth filtration (DF), one to three polishing chromatography steps are usually performed, however the sequence of the chromatography steps as well as the type of chromatography exist practically in all possible permutations (Shukla et al., 2007; Kelley et al., 2008a; Liu et al., 2019; Shukla et al., 2007). A flow-through chromatography step [Anion Exchange Chromatography (AEX) or Mixed Mode Chromatography (MMC)] is often optimized by increasing the pH to bind an increasing amount of impurities or by decreasing the conductivity to achieve the same goal (Kelley et al., 2008a; Kelley et al., 2008b). The next step is another bind-elute chromatography step, but this step often uses ionic interactions [Cation Exchange Chromatography (CEX)]. HCP removal can be optimised in a number of ways, one of the strongest being the use of gradient elution. This allows for a more selective elution and separation of impurities, which can often be beneficial also for aggregate removal (Ishihara and Yamamoto, 2005; Stein and Kiesewetter, 2007; Levy et al., 2016). Sometimes another type of chromatography, Hydrophobic Interaction Chromatography (HIC), is used instead of a CEX step or as a fourth chromatography step (Kramarczyk et al., 2008). The subsequent final downstream purification steps, such as virus filtration, ultrafiltration, diafiltration and finally formulation, often provide HCP levels as low as the detection limit of the ELISA assays used, making the relevant data more limited. If most of the hydrolytic activity originates from a single hydrolase, and the genomic knock-out or process optimization cannot be done or offer an improvement, the pH of the formulation can be slightly shifted to decrease the activity of the hydrolase, since they often have a pH optimum.

As described above, the removal of HCPs in the bioprocess is well understood, but specific information on the removal of the PS degrading HCPs is more limited. Specific characterisation work needs be carried out. Recombinantly expressed proteins and specific inhibitors have proven to be valuable in generating relevant knowledge, one of the earliest examples have been discussed describing LAL and LPL (Zhang et al., 2022b). For all these activities, the expression system and subsequent purification of the recombinant therapeutic protein must be carried out. Preferably, a CHO expression system should be used, and in most cases, the HCPs are expressed with protein tags that allow their specific capture. However, an ideal purification procedure should include different purification steps using different physico-chemical principles, to achieve a sufficiently high purity. A purification scheme consisting of affinity capture against the tag, complemented by at least high-resolution size exclusion chromatography (SEC), but ideally with a further separation technique based on charge. In line with this, Maier and colleagues used a variety of recombinantly expressed CHO hydrolases, which were tandem tagged to allow for an orthogonal purification to generate highly purified hydrolases (Maier et al., 2024a). The purified CHO hydrolases were then used for Downstream process characterisation studies, which revealed that similar elution behaviour of mAbs and specific CHO hydrolases during chromatographic separation represents the main cause of hydrolase contamination of mAb drug products. Thus, tailoring the polishing step based on the characterisation work led to the establishment of an optimized purification protocol that is able to separate mAbs and the contaminating HCPs more stringently (Maier et al., 2024a).

Another systematic characterisation work that was carried out earlier expressed a set of 20 PS-degrading enzymes (Kovner et al., 2023). After purification, the recombinant enzymes were tested for their ability to degrade PS, and a subset was subjected to more detailed characterisation, providing information on PS degradation kinetics and PS subspecies preference. In a very recent study, Maier and co-workers systematically expressed a list of 12 potentially PS degrading CHO hydrolases, which were identified in 7 different antibody formulations, in a LPL knockout CHO host cell line, followed by a multi-step purification process and comprehensive characterisation of the recombinant hydrolases (Maier et al., 2024b). characterisation studies with the purified hydrolases included analysis of cellular localization (by taking advantage of the native CHO DNA sequences), activity testing towards PS degradation and pH activity profiling. The authors revealed that while LIPA, PPT1, LPLA2, CES1, CES1F, CES2C, IAH1 and PAF-AH were confirmed to degrade polysorbate, GNS, SMPD1 and PLD3 did not show any signs of degrading activity. These results from Maier et al. enabled the establishment of a risk matrix of PS degrading CHO hydrolases with PAF-AH, LIPA, PPT1, and LPLA2 highlighted as most critical hydrolases based on their cellular expression, detection in purified antibody formulations, active cellular secretion, and PS degradation activity (Maier et al., 2024b). More of these studies would be needed to elucidate the specific characteristics of PS-degrading enzymes (see discussion above for more details).

PLBL2 was the first HCP to be identified in a drug product and was shown to be catalytically active and degrade PS (Dixit et al., 2016). However, a few years later, another paper (Zhang et al., 2020b) showed that PLBL2 was not actively degrading PS and that the previous activity could be attributed to another contaminating enzyme. Here, knock-out and immuno-depletion were used as a basis, and by quantitative analysis of PLBL2 in different drug products, no correlation between the amount of PLBL2 and the loss of PS was found. Mass spectrometry characterisation of recombinant PLBL2 revealed the presence of other HCPs, in particular LPLA2, and interestingly the degradation pattern was consistent with the available literature data on this enzyme. This example indicates clearly why rudimentary purification of recombinant enzymes are not necessary sufficient and higher purity should be strived for. Furthermore, for the identification of these proteins and also for recombinant expression, it is important to know the amino acid sequences used. Fortunately, regulatory authorities have also recognised this gap and the new US Pharmacopeia (USP) chapter on mass spectrometry (1132.1 chapter, Residual Host Cell Protein Measurement in Biopharmaceuticals by Mass Spectrometry) now includes not only peptides for identification and quantification, but also provides reference material in the form of recombinant proteins such as PLBL2.

These recombinant proteins should also be used to characterise their behaviour in the bioprocess and to generate specific knowledge on possible removal strategies. This will prove to be a powerful method to develop both general and process-specific solutions and mitigate many of the current problems. It is likely that these HCPs will not behave significantly differently from the rest of the HCP population. Alternatively, sensitive ELISA assays would also be very useful and could drive in-depth knowledge of the bioprocess to establish efficient removal strategies. The development of individual ELISA reagents is time consuming and costly. It is hoped that commercial HCP reagent suppliers will release specific assays for the critical HCPs involved in HCP degradation. The recombinantly expressed and purified lipases can also be utilized here, as they can be used in spiking studies and the tag should also allow high-throughput quantification.

Studies investigating the weak interactions between these HCPs and the antibody (Hecht et al., 2022) revealed binding sites at the CH1 domain that are conserved between IgG1 and 4 antibodies that bind PLBL2 and LPLA2. Glycosylation of the lipase was also shown to also play an important role. It is likely that protein engineering methods will be powerful to modify either the antibody or the protein sequence of the most critical lipases. However, if the interaction is weak, it is difficult to understand how some proteins still persist at the end of the bioprocess, when the standard process takes days and uses pH ranges from strongly acidic (virus inactivation, typically pH 3.2–3.8) to slightly basic (usually impurity wash step or similar flow-through chromatography steps) (Shukla et al., 2007). It is therefore reasonable to assume that these proteins behave, at least in part, similarly to antibodies in the polishing steps and that complete separation is probably not feasible. A valuable strategy may be to remove HCPs as early and as strongly as possible in the bioprocess to avoid possible breakthrough events. Furthermore, the assessment of the removal capacity of proteins based on their isoelectric point is highly simplified and more complex approaches may be required, such as the characterisation of recombinantly expressed critical HCPs in the bioprocess mentioned above.

In summary, in consideration of the current literature and the available process knowledge of the authors of this article, the probably most promising mitigation strategy to achieve substantially reduced PS degradation (regardless of the molecule format) includes the combined use of multi-hydrolase knockout CHO host cell lines and an optimized downstream purification platform process that can reliably remove remaining PS degrading hydrolases (which are, e.g., still present due to their essentiality for the CHO expression host). In this context, Maier et al. (2024b) recently presented a comprehensive characterisation of CHO host cell hydrolases and provided a risk matrix of the analysed enzymes, which might be used to guide future industrial bioprocess mitigation strategies towards development of particle-free drug products.

## Conclusion

In the past decade, evidence of enzymatic degradation of polysorbates (PS20 and PS80) has been reported extensively. As a result, in some cases product instability and/or the formation of sub- and visible fatty acid fatty acid particles have been reported.

As an alternative means to mitigating the enzymatic degradation of polysorbate, the use of alternative surfactants that lack an ester bond has been proposed (Morales et al., 2022; Ruiz et al., 2022; Roy et al., 2024). In order to achieve this, alternative surfactants must be capable of stabilising biologics to a similar extent as polysorbate 20/80. However, the majority of the tested surfactants have not been qualified and approved for parenteral applications. In a recent publication, Zürcher et al. (2024) put forth the hypothesis that the supplementary presence of L-arginine is necessary to mitigate the antibody aggregation that may be triggered by oleic acid release from polysorbate 80 (Zürcher et al., 2024).

As polysorbates can be degraded by different degradation mechanisms, it is important to identify the cause of surfactant degradation. To this end, a number of polysorbate analytical methods have been established and published in recent years to assess whether the surfactant is degraded by oxidation or by enzymatic degradation. The underlying opinion article reflects the ongoing discussions related to polysorbate degrading enzymes and focused on (i) the impact of polysorbate degradation on drug product quality attributes, (ii) analytical methods for identification, characterisation and quantification of polysorbate-degrading enzymes, (iii) enzyme activity, (iv) currently identified enzymes, and we further discussed (v) potential mitigation strategies that might eliminate enzymatic PS degradation during drug substance manufacturing in the future.

The identification of the root cause of polysorbate degradation is essential in order to develop meaningful mitigation strategies. In the event of potential enzymatic degradation, numerous mass spectrometric assays have been designed to detect and characterise the culprit enzyme(s) responsible for the degradation. The advent of new mass spectrometry technologies and enrichment strategies has significantly enhanced the identification of low abundance host cell proteins. This enhancement is crucial as enzymatically active proteins are capable of degrading polysorbate even at very low concentrations over weeks or months. Knowledge of the enzyme(s) responsible for polysorbate degradation allows different mitigation strategies to be tested, including the application of specially developed knock-out host cell lines, in which the critical enzymes have been genomically deleted, and/or the use of optimised manufacturing and purification processes. In the opinion of the authors, an impactful mitigation solution will likely rely on smart combinations of, e.g., tailored CHO knockout host cell lines with adapted downstream purification processes to enable the avoidance of polysorbate degradation in future bioprocesses.
